# Highly Active Ce- and Mg-Promoted Ni Catalysts Supported on Cellulose-Derived Carbon for Low-Temperature CO_2_ Methanation

**DOI:** 10.1021/acs.energyfuels.1c01682

**Published:** 2021-09-01

**Authors:** Pilar Tarifa, Cristina Megías-Sayago, Fernando Cazaña, Miguel González-Martín, Nieves Latorre, Eva Romeo, Juan José Delgado, Antonio Monzón

**Affiliations:** † Department of Chemical and Environmental Engineering, Instituto de Nanociencia y Materiales de Aragón (INMA), Consejo Superior de Investigaciones Científicas (CSIC)−University of Zaragoza, E-50018 Zaragoza, Spain; ‡ Department of Materials Science, Metallurgical Engineering and Inorganic Chemistry, University of Cádiz, E-11510 Puerto Real, Spain

## Abstract

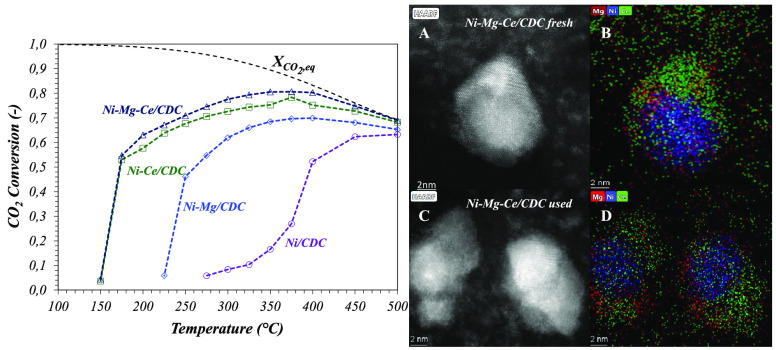

The CO_2_ methanation performance of Mg- and/or Ce-promoted Ni catalysts supported on cellulose-derived carbon (CDC) was investigated. The samples, prepared by biomorphic mineralization techniques, exhibit pore distributions correlated to the particle sizes, revealing a direct effect of the metal content in the textural properties of the samples. The catalytic performance, evaluated as CO_2_ conversion and CH_4_ selectivity, reveals that Ce is a better promoter than Mg, reaching higher conversion values in all of the studied temperature range (150–500 °C). In the interval of 350–400 °C, Ni–Mg–Ce/CDC attains the maximum yield to methane, 80%, reaching near 100% CH_4_ selectivity. Ce-promoted catalysts were highly active at low temperatures (175 °C), achieving 54% CO_2_ conversion with near 100% CH_4_ selectivity. Furthermore, the large potential stability of the Ni–Mg–Ce/CDC catalyst during consecutive cycles of reaction opens a promising route for the optimization of the Sabatier process using this type of catalyst.

## Introduction

Power to gas (PtG) technologies have emerged as a promising and sustainable solution to storage of renewable energy into chemical bond energy.^1^ Although hydrogen has a high energy density (9.7 MJ m^–3^ at 1.013 bar), the difficulties linked to its storage and transportation have motivated the pursuit of alternative technologies aiming to facilitate its manipulation, with its conversion into a gas fuel by CO_2_ methanation being a good example. Obtained methane has an even higher energy density (32.8 MJ/m^3^) and can be easily stored and distributed,^2^ and in addition, the process requires CO_2_ as reactive, which simultaneously emerges as an useful method to reduce CO_2_ emissions.

The global efficiency of these PtG plants, which must first entail CO_2_ capture and storage (CCS), strongly depends upon the methanation step.^3^ In this step, CO_2_ is hydrogenated to produce CH_4_ and water as byproducts (eq 1).
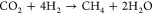
1The process is exothermic and, thus, thermodynamically favored at low reaction temperatures (Δ*H*_r,298 K_ = −164.6 kJ/mol and Δ*G*_r,298 K_ = −113.2 kJ/mol) and has a kinetic barrier that involves a complex eight-electron reduction process.^4^ Unfortunately, it is accompanied by side reactions, like the reverse water–gas shift (RWGS, eq 2) and CO methanation (eq 3), which strongly compromise the performances toward methane.
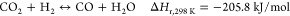
2
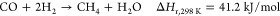
3Thermodynamic analysis of the transformation of CO_2_ into CH_4_ indicates that CO_2_ conversion is minimum at 600 °C and that CH_4_ selectivity might be 100% at temperatures below 400 °C.

The development of active, selective, and stable catalysts for the CO_2_ methanation reaction has been a challenge in the last decades.^5^ In 1975, Vannice^6^ determined that the activity of CO methanation increases in the order Ru > Fe > Ni > Co > Rh > Pd > Pt and Ir. Considering the price and observed catalytic performance, Ni-based catalysts have been commonly used in the CO and CO_2_ methanation reactions; their main drawback has been the fast deactivation caused by coke deposition, the sintering of the particles, and/or the catalyst structural changes.^7^ Nevertheless, the performance and lifetime of the catalyst depend largely upon the synthesis method, the used support, the dispersion of the active phase, the metal–support interactions, and the presence of promoters.^8,9^

Metal oxides, such as α-Al_2_O_3_, TiO_2_, MgO, ZrO_2_, or CeO_2_, have been the most commonly used supports.^2,9−12^ Among them, CeO_2_ was particularly interesting and active, especially at low reaction temperatures. The latter has been related to its redox properties and the presence of oxygen vacancies (as a result of the co-existence of the Ce^4+^/Ce^3+^ redox pair), which favor the stronger interaction with the active phase and, consequently, the good dispersion of the particles.^13^ In addition to this, the oxygen vacancies may act as adsorption sites for CO_2_ molecules.^14,15^ On the other hand, CO_2_ adsorption and activation are enhanced by the presence of basic sites, thus improving its conversion to CH_4_;^14,16^ this effect was observed over MgO.^5^

Other novel materials, such as structured catalysts,^17,18^ zeolite,^19^ or carbon supports,^20−22^ have also been studied. Among them, carbon materials show unique properties, such as a high surface area, high thermal conductivity, hydrophobic character, and high hydrogen storage ability,^23−25^ which could be highly beneficial for CO_2_ methanation to well disperse the active phase, to avoid hot spots, to facilitate water removal, and/or to supply hydrogen to the active phase. These materials can be obtained sustainably from renewable and abundant resources, like biomass.^26^ Using biomorphic mineralization techniques, biomass used as a biotemplate is thermally decomposed under reducing conditions, obtaining a carbon material that preserves the morphology of original biomass.^27^ In this sense, it is possible to obtain materials with a hierarchically complex structure, inherited from biomass, whose final structure will logically depend upon the raw material used. The properties of the final carbon can be likewise tuned by varying the conditions of the thermal decomposition (temperature, heating rate, or time, among others).^28^

Even more interestingly, biomass might be impregnated with metallic precursors prior to the thermal decomposition in a way that it is possible to obtain a one-pot catalyst with metal nanoparticles highly dispersed on the carbonaceous support.^29,30^ This synthetic approach offers the possibility to finely tune and design the catalyst, which can be thus optimized for several applications,^23^ highly reducing costs. In this context, our group has developed these kind of carbonaceous-based catalysts using mainly lignocellulosic materials as a carbon precursor for several catalytic applications that include synthesis of carbonaceous nanomaterials by catalytic chemical vapor deposition (CCVD),^31−33^ liquid-phase hydrogenations,^34,35^ and hydrodeoxygenation reactions.^36,37^

Moving a step forward in this work, Ni-based catalysts supported on cellulose-derived carbon have been synthesized by biomorphic mineralization techniques and tested in the CO_2_ methanation reaction. Considering the beneficial effects of MgO and CeO_2_ in the mentioned reaction, both species have been included as Ni promoters (Mg and/or Ce). The catalysts have been deeply characterized, and these results are related to their catalytic activity. The potential stability of the optimized catalyst was also evaluated.

## 2 Experimental Section

### 2.1 Catalyst Synthesis

All catalysts were prepared by the incipient wetness impregnation of cellulose with aqueous solutions of the metallic precursors [Ni(NO_3_)_2_·6H_2_O from Alfa Aesar, Mg(NO_3_)_2_·6H_2_O from Panreac, and Ce(NO_3_)_3_·6H_2_O from Sigma-Aldrich]. Ni loading was fixed to 3.5 wt % with respect to the raw cellulose (dried, before thermal decomposition) as well as Mg and Ce promoters with the atomic ratios of Ni/Mg = 1:1, Ni/Mg/Ce = 1:0.5:0.5, and Ni/Ce = 1:1. After impregnation, the samples were placed in a horizontal tubular oven and dried at 80 °C overnight. The catalysts were obtained after thermal decomposition of the samples at 600 °C for 3 h under a reducing atmosphere (50% H_2_, 50% N_2_, and 300 mL/min total flow), with a fixed heating rate of 50 °C/min. Samples were cooled in inert gas (150 mL/min N_2_ flow) and then passivated by flowing a CO_2_/N_2_ mixture (16% CO_2_ and 84% N_2_) for 1 h. The samples were named according to the metal composition as Ni–Mg/CDC, Ni–Mg–Ce/CDC, and Ni–Ce/CDC, with CDC being used to denote the support, i.e., cellulose-derived carbon.

### 2.2 Characterization Techniques

The textural and structural properties of the catalysts were determined by X-ray diffraction (XRD) analysis, thermogravimetric analysis (TGA), N_2_ adsorption measurements, transmission electron microscopy (TEM), and high-angle annular dark-field scanning transmission electron microscopy (HAADF-STEM).

XRD analysis was performed in a Siemens D-5000 (45 kV and 40 mA) diffractometer equipped with a Cu anode (Kα radiation, λ = 0.1542 nm). The diffractograms were acquired in a continuous scan mode from 5° to 90° 2θ with 0.02° counting step and a step time of 4 s. The structural determination was carried out through the pattern comparison using an International Centre for Diffraction Data (ICDD) database and High Score Plus (PANalytical) software. The crystallite size of each species was calculated using the Scherrer equation.

The thermal stability of the samples was evaluated in air (50 mL/min flow) using a Mettler Toledo STA/SDTA 851e thermogravimetric instrument. The measurements were likewise used to estimate the catalyst composition assuming that the carbonaceous support is totally burned and the metals are fully oxidized to NiO, MgO, and CeO_2_. The calculations were made considering the metallic atomic ratio in the samples (Ni/Mg = 1:1, Ni/Ce = 1:1, and Ni/Mg/Ce = 1:0.5:0.5) and the initial/final weight of the samples.

Metal loadings were obtained through atomic absorption spectrometry (AAS) using the SpectrAA 110 instrument from Varian, Inc. Around 10 mg of sample was diluted in a mixture of HCl and HNO_3_ (4:1) with a total volume of 100 mL and stirred for 3 h. After filtration, the absorbance of the liquid was measured using an air–acetylene flame in a wavelength of 202.6 and 232 nm to measure Mg and Ni, respectively. Given that Ce cannot be measured by AAS, the content has been estimated considering the nominal atomic metallic ratio of the elements.

The reducibility of catalysts was evaluated by means of the temperature-programmed reduction using H_2_ (H_2_-TPR) and in a ChemBet PULSAR Quantachrome equipment. A total of 50 mg of sample was loaded in the reactor, dried under Ar flow at 120 °C for 1 h, and finally cooled to room temperature. After that, the reduction was carried out using 20 mL/min of a 10% H_2_/Ar mixture and the temperature was increased to 750 °C with a heating rate of 10 °C/min. The thermal conductivity detector (TCD) signal was acquired in a continuous mode.

Acid–base properties of fresh catalysts were evaluated through temperature-programmed desorption of CO_2_ (CO_2_-TPD) using an AUTOCHEM II 2920 (Micromeritics) instrument equipped with a TCD. The samples were first dried in He (50 mL/min flow) at 600 °C for 1 h and then cooled at 80 °C, using a 15 °C/min heating rate in both cases. CO_2_ adsorption was ensured by flowing 50 mL/min of a 10% CO_2_/Ar mixture for 1 h. The desorption curve was then obtained by increasing the temperature at 10 °C/min until 600 °C under an Ar atmosphere (50 mL/min flow).

The carbonaceous structure of fresh and spent catalysts was characterized by Raman spectroscopy using a WiTec Alpha300 confocal Raman microscope with a 532 nm laser excitation beam. Five Raman spectra were acquired from different spots of each sample in the Raman shift range of 50–3400 cm^–1^ to visualize metal oxides (300–700 cm^–1^) and the characteristic bands of carbonaceous materials: D (∼1350 cm^–1^), G (∼1580 cm^–1^), and 2D (∼2690 cm^–1^).

The chemical composition of the surface was characterized by X-ray photoelectron spectroscopy (XPS) in a Kratos Axis ULTRA spectrometer using non-monochromatic Al Kα radiation (*h*ν = 1486.7 eV). All spectra were analyzed using CASA XPS software by applying a Shirley-type background.

The specific surface areas and pore distributions were obtained from the N_2_ adsorption–desorption isotherms carried out at 77 K in Tristar 3000 (Micromeritics Instrument Corp.) equipment. The specific areas were calculated according to the Brunauer–Emmett–Teller (BET) method, whereas the total pore volume and average pore diameter were obtained according to the Horváth–Kawazoe method. The *t*-plot method was also used to determine the micropore volume. Additionally, pore size distributions were obtained by non-local density functional theory (NLDFT) calculations.

TEM micrographs were acquired in a FEI Tecnai T-20 microscope operating at 200 kV. The metal particle size distributions were estimated by counting at least 500 nanoparticles/sample and using ScopePhoto 3.0 software. A more detailed high-resolution analysis (HAADF-STEM) of Ni–Mg–Ce/CDC was carried out in a FEI Titan Cube Themis 60–300 microscope (Cs = 0.001 mm and sub-angstrom resolution) operating at 200 kV with a camera length of 11.5 cm and aberration correction of 60–300. This microscope also offers a Super X-G2 option in STEM mode, which was employed to acquire STEM images of this catalyst at 200 pA and dwell time per pixel of 128 μs. Energy-dispersive X-ray spectroscopy (X-EDS) mapping was performed, aiming to elucidate the distribution of each element. Maps were filtered using Gaussian blur of 0.8, using Velox software to improve their visual quality.

### 2.3 Catalytic Tests

The hydrogenation of CO_2_ was carried out in a vertical fixed-bed tubular (inner diameter of 7 mm) reactor at atmospheric pressure and temperatures ranging from 150 to 500 °C. In a typical experiment, we would load the catalyst (50–100 mg) in the reactor fixed with a quartz wool plug. The exact mass would then be properly adjusted to maintain a constant CO_2_/Ni ratio in all of the experiments (CO_2_ molar flow/Ni mass would be set to 3.61 mol of CO_2_ g^–1^ of Ni h^–1^). Prior to the reaction, the catalysts were reduced “*in situ*” at 500 °C for 1 h under a total flow of 400 mL/min (50% H_2_ and 50% N_2_). Then, the reaction was carried out introducing a controlled mixture of H_2_/CO_2_/N_2_ with a stoichiometric ratio of 4:1:2. The total flow (185 ± 10 mL/min) was determined considering the fixed *F*_CO_2__/*m*_Ni_ value. In these operating conditions, the spatial velocity was high enough [gas hourly space velocity (GHSV) ranging from 30 × 10^3^ to 38 × 10^3^ h^–1^] to properly evaluate the intrinsic activity of the catalysts.

The reacted mixture flowed through a Peltier condenser to remove water and was finally analyzed by gas chromatography using N_2_ as the internal standard in an Agilent MicroGC 490 equipped with two columns (Molsieve 5A and Poraplot Q). The catalytic activity was measured every 25 °C in the 150–500 °C temperature range, with the plotted conversion and selectivity values corresponding to the average of five injections at least (30 min in each temperature). As for the stability test, two cycles were carried out in isothermal conditions (250 °C), with the outlet gases being analyzed every 5 min. The obtained carbon balances were 100 ± 5% in all measurements. Conversion and selectivity were calculated according to eqs 4 and 5.
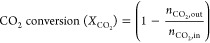
4
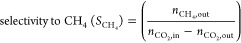
5

## 3 Results and Discussion

### 3.1 Characterization of Fresh Catalysts

The thermal stability of the samples was evaluated in air, and the results are presented in Figure SI-1 of the Supporting Information. A common slight weight loss is observed in all samples around 80 °C, which is ascribed to the loss of physisorbed water. This initial loss is lower in the bare support in comparison to the catalysts as a result of the higher hydrophobicity of the carbonaceous support.^25^ In addition, the addition of metals introduces O^–^ and OH^–^ groups associated with CeO_2_ and MgO to change the chemical surface of the carbonaceous support. The main weight loss, which takes place between 200 and 460 °C depending upon the composition of the material, is attributed to both the oxidation of metallic Ni nanoparticles and the combustion of the support. The catalytic effect of the metals in the combustion process is clearly evidenced, with the combustion temperature of the catalysts being lower than that of the bare support.^38^ The increase in the Ce content leads to a decrease in the combustion temperature, which suggests a higher catalytic effect of Ce in oxidizing environments in comparison to Mg. This fact might be related to the presence of oxygen vacancies in the CeO_2_ lattice as a result of the Ce^4+^/Ce^3+^ redox couple,^39^ which boosts the fast oxygen exchange with the environment, thus also facilitating the carbon combustion at lower temperatures. Indeed, the combustion temperature is directly related to Ce contents in the final solids: the higher the atomic percentage (Ni–Ce/CDC > Ni–Mg–Ce/CDC > Ni–Mg/CDC > CDC), the lower the combustion temperature. Nevertheless and to a lesser extent, the presence of Ni and Mg (Ce, 0 atomic %) also affects the support thermal stability, decreasing the combustion temperature with respect to bare CDC as a result of the metal catalytic effect.^38^

The experimental metal loadings were estimated from both TGA-air and atomic absorption spectroscopy (AAS), following the method described in the Experimental Section. For the TGA-air results, the total combustion of the support and the complete oxidation of the metals (NiO, MgO, and CeO_2_) have been assumed. For the calculations, the nominal atomic ratios of the metals in the samples (Ni/Mg = 1:1, Ni/Ce = 1:1, and Ni/Mg/Ce = 1:0.5:0.5) were also considered (Table 1). As seen in Table 1, the results are quite similar for both techniques, confirming a high metal content in the final catalysts. The final metal loadings are logically much higher than the initial metal loadings (fixed and calculated on a dried cellulose basis) as a consequence of the cellulose decomposition during the synthesis, which leads to the weight loss in the form of vapors and organic liquids. Ni–Mg/CDC has the highest Ni content, followed by Ni–Ce/CDC and Ni–Mg–Ce/CDC samples, which present a similar percentage, suggesting that the decomposition of cellulose is major in the presence of Ni and Mg. Besides, it can be observed that the measured Mg and Ce weight contents of the bimetallic catalysts (Ni–Mg/CDC and Ni–Ce/CDC) are very different (ca. 16 versus 40 wt % Mg and Ce, respectively), although the metallic atomic ratios (Ni/metal = 1:1) are the same for these catalysts. This is due to the high difference between Mg and Ce molar weights (Mg = 24.3 g/mol and Ce = 140.1 g/mol). With regard to the CDC weight percentage, Ni–Mg–Ce/CDC presents a higher CDC content and, as a consequence, lower total metal weight percentage.

**Table 1:** Nominal Atomic Ratios, Nominal Ni (wt %), and Experimental Composition Values Determined from TGA-Air and AAS

catalyst	initial Ni (wt %)	Ni/Mg/Ce (atomic ratio)	Ni^a^^/^b (wt %)	Mg^a^^/^b (wt %)	Ce^a^^/^b (wt %)	CDC^a^ (wt %)
CDC						99.5
Ni–Ce/CDC	3.5	1:0:1	17:17.8		40:41.0	43
Ni–Mg–Ce/CDC	3.5	1:0.5:0.5	17:17.5	4:3.6	21:20.7	58
Ni–Mg/CDC	3.5	1:1:0	38:37.6	16:16.6		46

a Metal weight percentage calculated from TGA-air.

b Metal weight percentage obtained from AAS.

**Figure 1:**
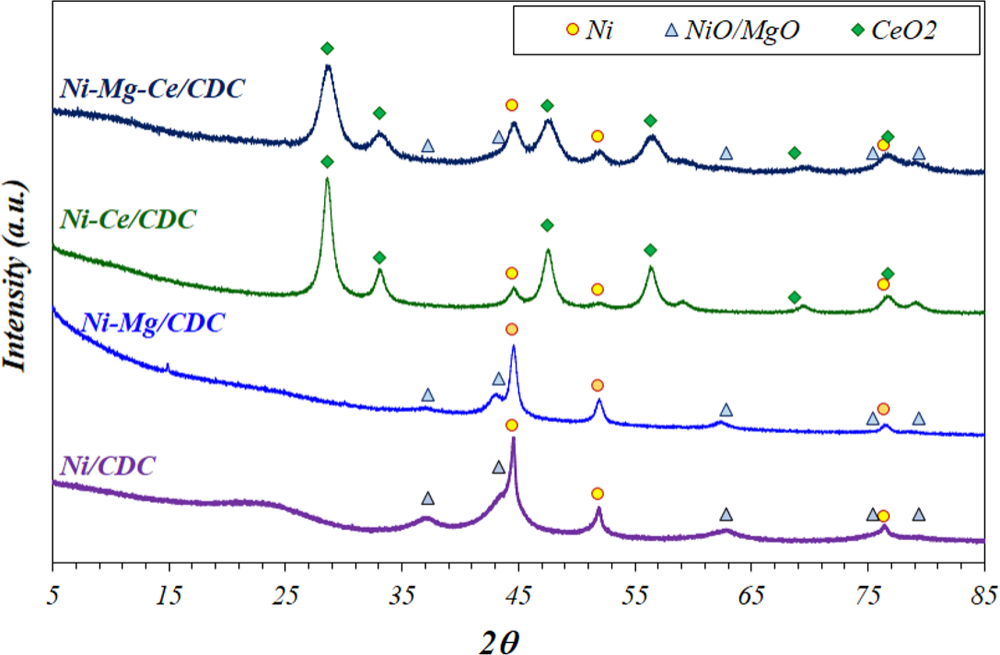
XRD diffractograms of fresh catalysts.

The XRD diffractograms of fresh catalysts and bare CDC support are presented in Figure 1. In general, the XRD patterns of biochars are mostly amorphous, showing signals at around 26.2°, 42.2°, and 77.1° 2θ, which correspond to (002), (100), and (110) planes, respectively. The presence of Ce oxide (CeO_2_) and Ni^0^ species is clearly evidenced in the catalysts. The shape and intensity of diffraction peaks suggest their existence as nanoparticles of low diameter. It is worth pointing out that Ni nanoparticles remain partially reduced after the synthesis as a consequence of the reducing atmosphere used. This fact clearly facilitates the total activation/reduction of the catalysts before the catalytic tests. On the Ni–Mg/CDC sample, the signals appearing around 42.9°, 62.3°, 74.7°, and 78.7° 2θ could correspond to NiO and/or MgO, almost being impossible to accurately specify which one is which as a result of the similarity of the XRD patterns.^40^ Therefore, NiO could co-exist with metallic Ni in the samples.

The average particle sizes calculated through the Scherrer equation are summarized in Table 2. The Ni–Mg/CDC sample shows the biggest Ni particle size, with an average value of 21 nm, whereas replacing Mg with Ce results in the Ni particle size drop until 10 nm. This drop is attributed, on the one hand, to the presence of ceria with vacancies of oxygen, which can interact with metallic Ni nanoparticles preventing its sintering^13,14^ and, on the other hand, to the higher amount of Ni observed in the Ni–Mg/CDC sample, which facilitates its sintering during the preparation step. Partial replacement of Mg with Ce in the Ni–Mg–Ce/CDC sample results in the smallest Ni particle size, 8 nm. As for CeO_2_ and MgO particle sizes, both are smaller than Ni in all cases, with values ranging from 4 to 8 nm.

**Table 2:** Average Particle Sizes of Fresh and Used Catalysts Obtained from the XRD and TEM

catalyst	Ni^0^ a (nm)	NiO/MgO^a^ (nm)	CeO_2_^a^ (nm)	*d*_particle_^b^ (nm)	*D*^c^ (%)
Ni/CDC	27:65	6:–			3.7
Ni–Ce/CDC	10:9		8:6	8.0 ± 7.3	12.5
Ni–Mg–Ce/CDC^e^	8:6:7	5:6:5	4:4:5	9.0 ± 5.6	10.0
Ni–Mg/CDC	21:34	6:5		18.8 ± 45.4	4.8

a Average particle size calculated using the Scherrer equation, taking the peaks at 44.5° for Ni^0^, 28.6° for CeO_2_, and 43.3° for NiO or MgO.

b Average value and standard error of the particle size distribution obtained by TEM.

c Ni dispersion calculated as 1/*d*_pNi_, with *d*_pNi_ being the Ni particle size obtained from XRD.

Nevertheless, some of the observed differences are not that significant when the size values obtained from TEM are also considered (Table 2). The larger particle size in the Ni–Mg/CDC sample is undeniable; however, the average particle size differences observed in Ce-containing samples are negligible if both estimation methods are considered. In this case, the differences seem to be related to the size distribution, narrower in the case of the Ni–Mg–Ce/CDC catalyst, as indicated in the variance values. Therefore and even though the Ce content in this catalyst is lower in comparison to the Ni–Ce/CDC sample, the particle sizes are more homogeneous in the presence of a certain Mg amount, which somehow would denote a kind of synergism between Mg and Ce during the synthesis process.

**Figure 2:**
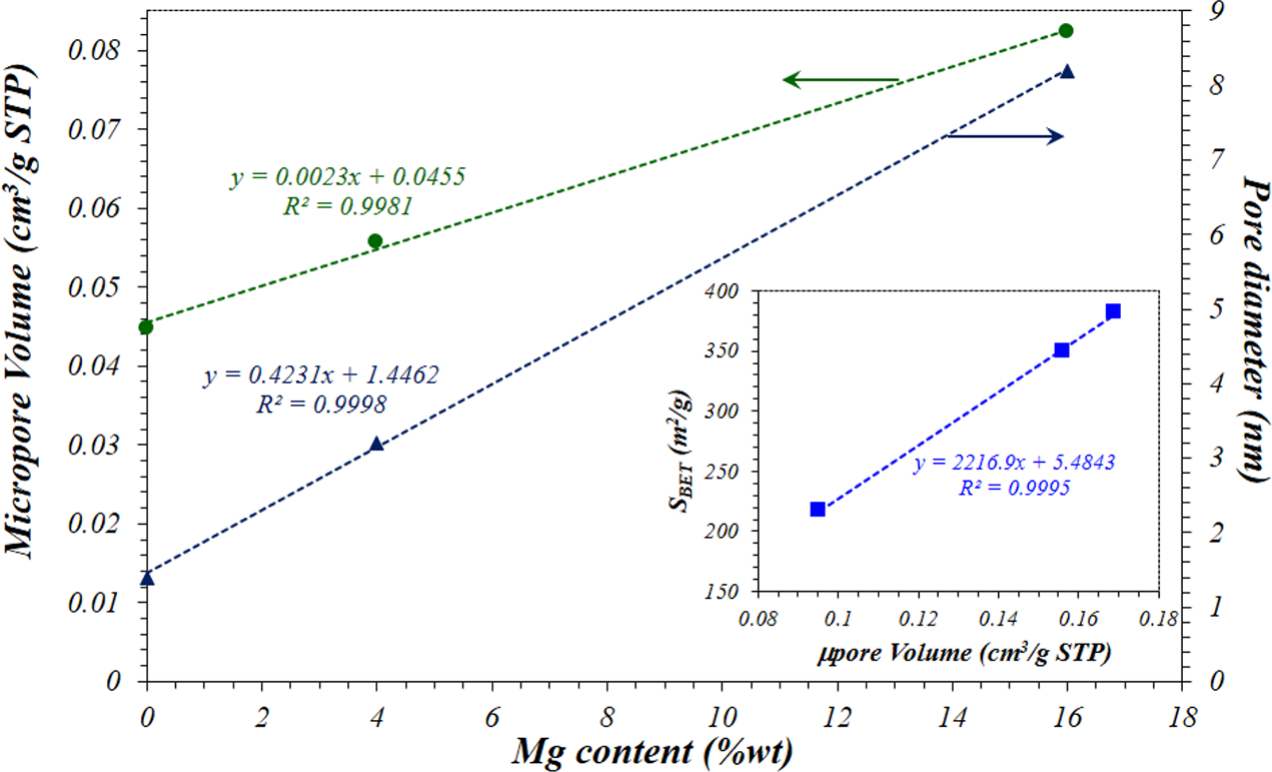
Dependence of the micropore volume and pore diameter upon the Mg content. (Inset) *S*_BET_ versus micropore volume.

The textural properties of bare CDC and the catalysts are compared in Table 3. CDC presents a microporous structure with a high surface area, which is characteristic of this type of carbonaceous material.^23^ All catalysts have a lower specific surface area than the support, accompanied by a drop in the micropore percentage/micropore volume along with an increment in the average pore diameter. Besides, these changes are more pronounced on Mg containing solids. The higher the Mg content, the higher the pore volume and pore diameter and the lower the microporous percentage. These trends can be observed in Figure 2, where the micropore volume and pore diameter are represented versus the Mg weight percentage in the catalyst. As observed, both variables increase linearly, having large correlation coefficients, which entails a severe decrease in microporosity for the Ni–Mg/CDC sample. The linear relationship between the BET surface area and the micropore volume is also included (inset of Figure 2).

**Table 3:** Textural (N_2_ Adsorption) and Basic (CO_2_-TPD) Properties of the Fresh Catalysts

catalyst	*S*_BET_ (m^2^/g)	pore volume (cm^3^/g)	micropore volume (cm^3^/g)	micropores (%)	*d*_pore_ (nm)	μmol of CO_2_/g of metal
CDC	457	0.21	0.150	90	0.7	
Ni–Ce/CDC	217	0.18	0.045	53	1.4	187
Ni–Mg–Ce/CDC	350	0.39	0.056	40	3.2	713
Ni–Mg/CDC	382	0.49	0.082	15	8.2	737

These linear correlations have been previously reported for carbonaceous materials^41^ and metal-supported cellulose-derived carbons.^35^ Thus, the slope of *S*_BET_ versus micropore volume obtained in the present case, 2217 m^2^/cm^3^, is quite similar to those obtained by Scherdel et al.,^41^ 2559 m^2^/cm^3^, and Cazaña et al.,^35^ 2659 m^2^/cm^3^. This value is mainly determined by the temperature and time of the carbonization stage.^35,41^

**Figure 3:**
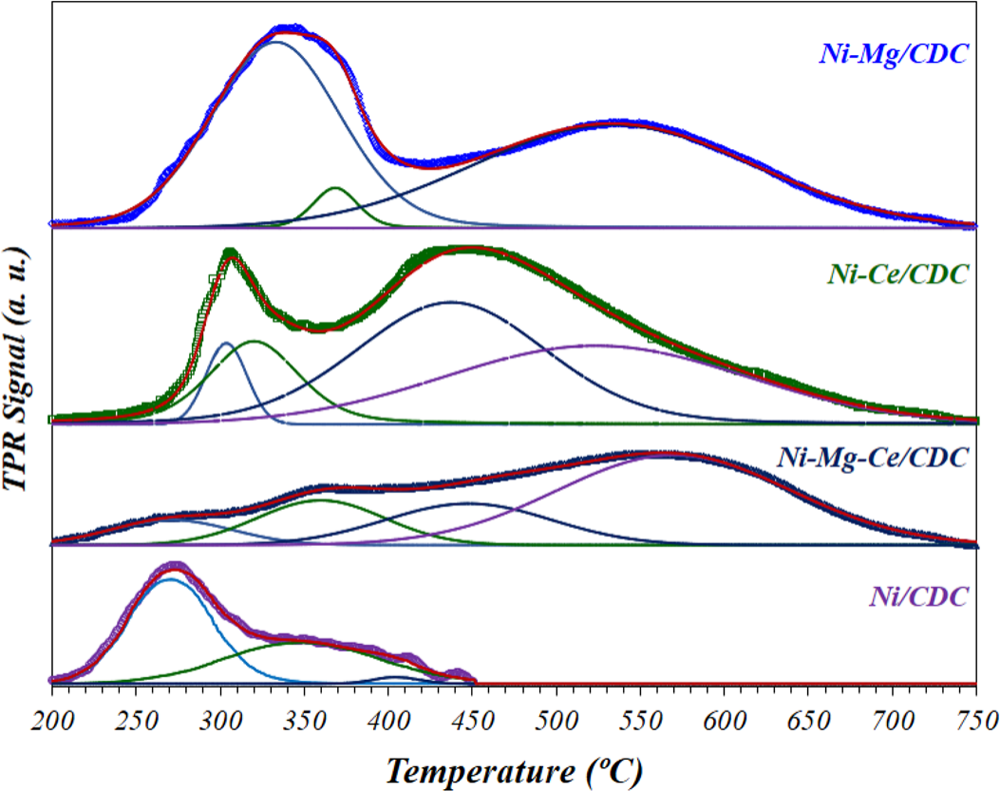
H_2_-TPR profiles of the fresh catalysts.

The reducibility of the catalysts was evaluated by H_2_-TPR experiments (Figure 3). It should be noted that both NiO and CeO_2_ are the predominant reducible species in those samples. The H_2_-TPR profile of the Ni/CDC sample shows only one peak, corresponding to the reduction of NiO species supported on the carbonaceous material.^42,43^ The broadness of this peak, having at least the contribution of two processes (with maximums at 275 and 350 °C), is probably the consequence of the wide particle size distribution of this catalyst (see Figure 5A). The presence of Mg and/or Ce modifies the reducibility of the catalysts, as a consequence of the different interactions between the metals at the surface. In this sense, the Ni–Mg/CDC catalyst exhibits two peaks: one peak at a low temperature (340 °C) and a second peak at a higher temperature (550 °C). These peaks are attributed to the reduction of NiO weekly interacting with MgO and the reduction of NiO species in a strong interaction with MgO surface defects, respectively.^44^ A similar behavior is observed for the Ni–Ce/CDC catalyst, showing two reduction zones centered at ca. 305 and 450 °C, respectively. The low-temperature peak can be likewise assigned to the reduction of Ni^2+^ weekly interacting with ceria, whereas the second reduction zone could be partially attributed, on the one hand, to the NiO species having a strong interaction with Ce^4+^ species and, on the other hand, to the reduction of ceria.^45^ Finally, the Ni–Mg–Ce/CDC catalyst show a low-temperature reduction zone below 425 °C, composed of two wide peaks at 260 and 370 °C and a high-temperature reduction zone with broad peaks at 450 and 570 °C, respectively. The lower temperature peak (260 °C) can be attributed to the reduction of highly dispersed and low-sized NiO nanoparticles,^46^ and in a similar way to the Ni–Ce/CDC sample, the peaks at 370 and 450 °C might be assigned to Ni^2+^ weakly and strongly interacting with ceria, respectively, with the peak at 570 °C corresponding to ceria reduction.

**Figure 4:**
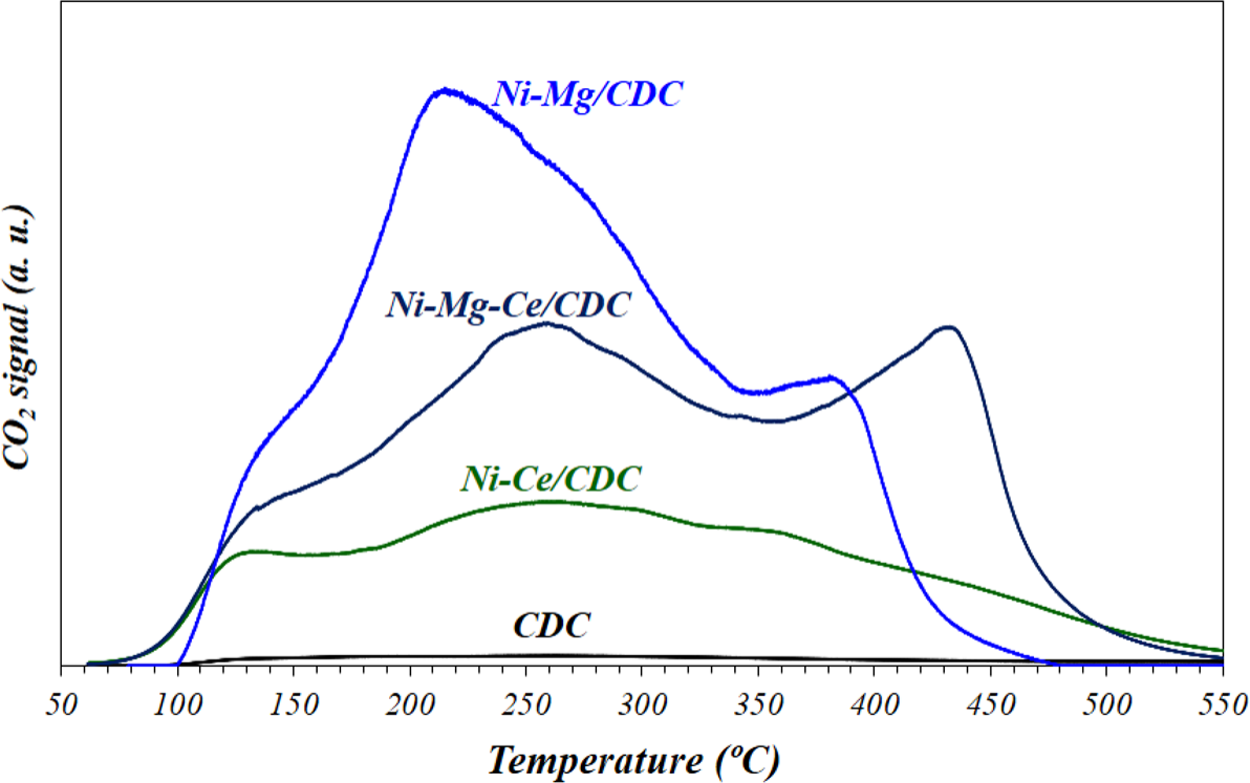
CO_2_-TPD profiles of the fresh catalysts.

The strength and distribution of the basic sites in the fresh samples were studied by CO_2_-TPD. As observed in Figure 4, the distributions of weak (below ca. 180 °C), moderate (until 350 °C), and strong (above 350 °C) basic sites are different for each catalyst.

For comparison, the CO_2_-TPD experiment of the bare carbon support is presented, showing the absence of this type of site and indicating that the basic character is given by the presence of Ni, Mg, and Ce. The quantification of these TPD profiles, expressed as micromoles of CO_2_ adsorbed per gram of metal, gives the values shown on Table 3, which follows the order: Ni–Mg/CDC > Ni–Mg–Ce/CDC > Ni–Ce/CDC. Thus, the number of basic sites in Mg-containing samples is almost 4 times higher than that of the Ni–Ce/CDC catalyst, which is congruent with the basic nature of Mg oxide. However, as in the H_2_-TPR results, the distribution of sites is also different. In this sense, only Mg-containing samples present a peak above 370–380 °C, indicating that the presence of strong sites is directly related to the presence of Mg^2+^ species on the surface.

The results published in the literature are controversial with respect to which type of basic site is the most favorable for CO_2_ methanation.^47^ Some authors claims that moderate adsorption sites are pivotal for CO_2_ methanation, while both weak and strong sites are inactive.^48,49^ However, other authors indicate that stronger basic sites are necessary to enhance the CO_2_ absorption and activation.^50,51^ Contrary, the enhancement of weak basic sites has also been claimed.^52^ In any case, a higher basicity is necessary to improve the catalyst performance, although this factor is not the only factor responsible for the enhancement of the activity; other factors, like metal dispersion and metal–support interaction, can also affect the enhancement of the activity.

Raman spectra of fresh and used samples (panels A–C of Figure SI-2 of the Supporting Information) show the presence of the typical D and G bands at 1350 and 1590 cm^–1^ respectively, characteristic of the carbonaceous support. These bands are similar for all of the samples, not changing after the reaction. Furthermore, in the zone from 300 to 700 cm^–1^ (Figure SI-2C of the Supporting Information), several bands corresponding to the presence of NiO, CeO_2_, and/or MgO can be observed. The band at ca. 460 cm^–1^ in Ce-containing catalysts is assigned to the F2g vibration of the fluorite lattice of ceria.^53^ The broad bands between 490 and 570 cm^–1^ are related to the disorder-induced one-phonon scattering characteristic of NiO and MgO oxides.^54,55^ The simultaneous presence of Ce and Mg decreases the Raman signal in this region as a result of the difference in their ionic radio; see spectra of Ni–Mg–Ce/CDC in Figure SI-2C of the Supporting Information, as previously reported by Siakavelas et al.^45^

In addition, the fresh catalysts were also characterized by XPS, with the results being presented in Table SI-1 and panels A–D of Figure SI-3 of the Supporting Information. These results reveal the presence of low quantities of reduced Ni in the fresh samples as a consequence of the passivation stage carried out during the preparation protocol; otherwise, the catalysts would be totally reduced but highly unstable as a result of the pyrophoric character of the nickel nanoparticles.

**Figure 5:**
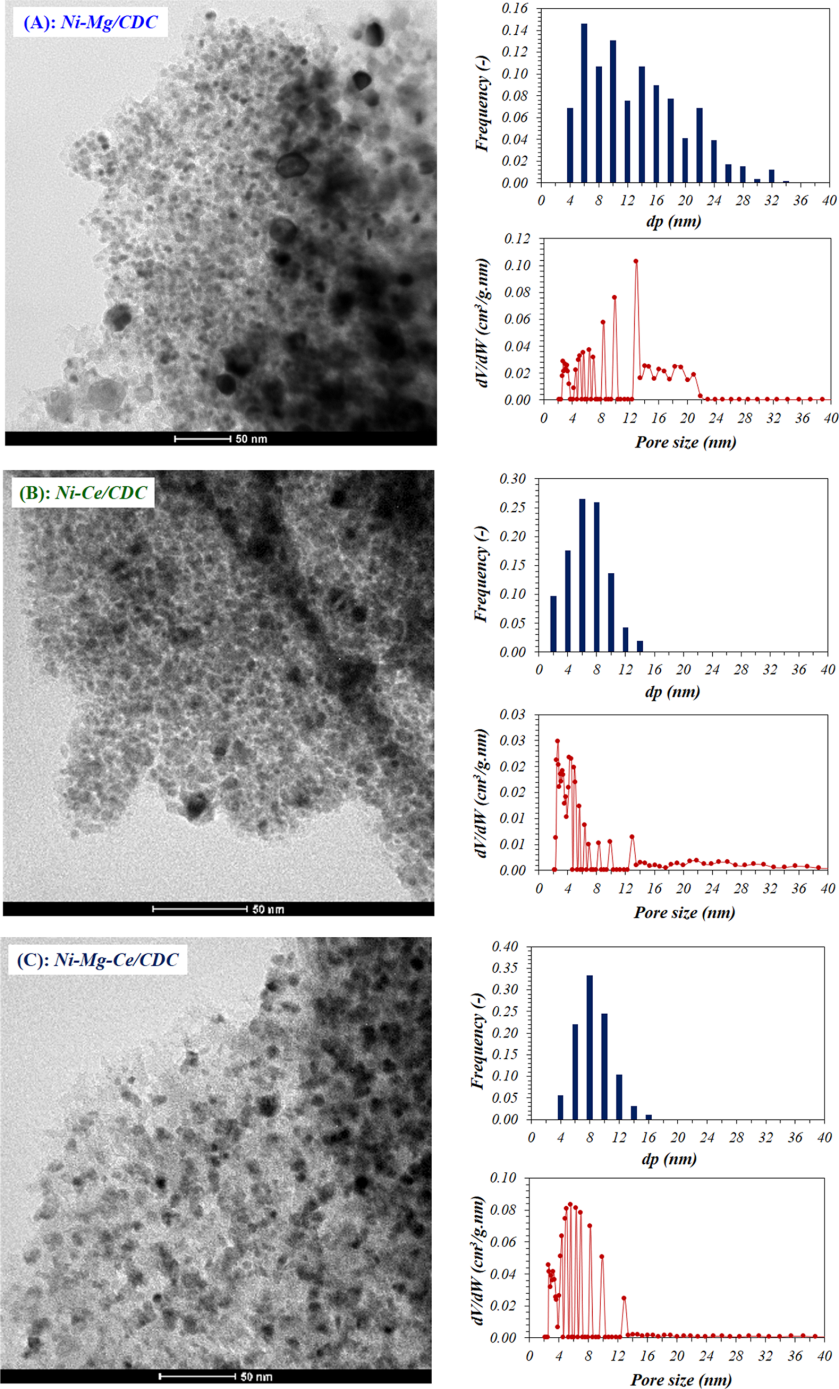
Representative TEM images of fresh catalysts, particle size distributions, and differential pore volumes (NLDFT) of fresh catalysts: (A) Ni–Mg/CDC, (B) Ni–Ce/CDC, and (C) Ni–Mg–Ce/CDC.

**Figure 6:**
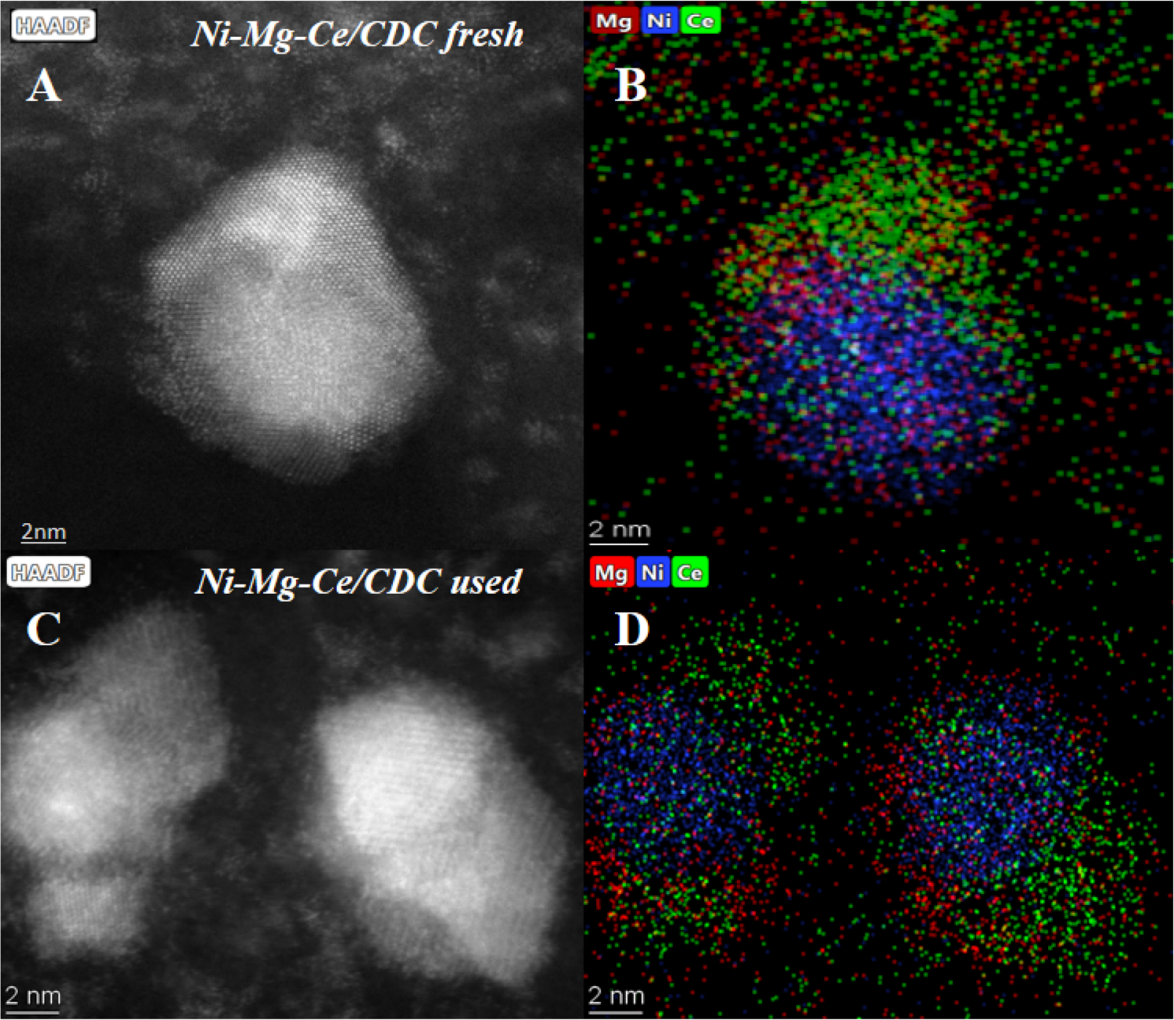
HAADF images and X-EDS maps of (A and B) fresh and (C and D) used Ni–Mg–Ce/CDC catalysts.

The dispersion of particles and size of fresh catalysts were carefully analyzed by TEM (Figure 5) and HAADF-STEM (Figure 6). Figure 5 displays representative TEM pictures (left), along with the corresponding frequency histograms (top right) and the differential pore volumes (bottom right), obtained through NLDFT simulation from N_2_ adsorption isotherms. The images show well-dispersed particles on the carbonaceous support, also confirmed in the histograms, likewise indicating that particles are in the nanoparticle range, with average diameters being in good agreement with the XRD results of the fresh samples (see Table 2). As commented above, the introduction of Ce in the catalysts clearly leads to smaller particle sizes and narrower size distributions, which results in a better dispersion of the particles over the support. This fact is linked to the well-known capacity of Ce to prevent particle sintering^14^ and the higher Ni content of the Ni–Mg/CDC catalyst, as mentioned previously. These images indicate an excellent interaction, in both fresh and used samples, among Ni, Ce, and Mg, which is probably responsible for the high activity of this catalyst.

Furthermore, the differential pore volume distributions are also presented in Figure 5 (bottom right). In comparison of these results to those obtained for the particle size distributions (top right in Figure 5), we found that, interestingly, the shape of both curves is similar in all cases, which suggests that the pore diameter is related to the metal particle size, especially in Mg-containing catalysts. It seems that the cellulose thermal decomposition process is directed by the metallic nanoparticles, and the porous structure (mainly the pore size distribution) of the prepared carbon is determined by these metallic nanoparticles. In this way, for instance, Ni–Mg/CDC shows the highest average particle size, which is in concordance with the lowest microporosity found by N_2_ adsorption, 15% (see Table 3).

With regard to the surface atomic ratios determined by XPS (Table SI-1 of the Supporting Information), an enrichment of Mg and a decrease of the Ce content are observed with respect to the nominal Ni concentration. It should be considered that these values are averaged for the entire surface; however, the local concentration of each specific metallic nanoparticle (NP) is different, as shown by the HAADF and X-EDS results presented in Figure 6.

### 3.2 Catalytic Tests

**Figure 7:**
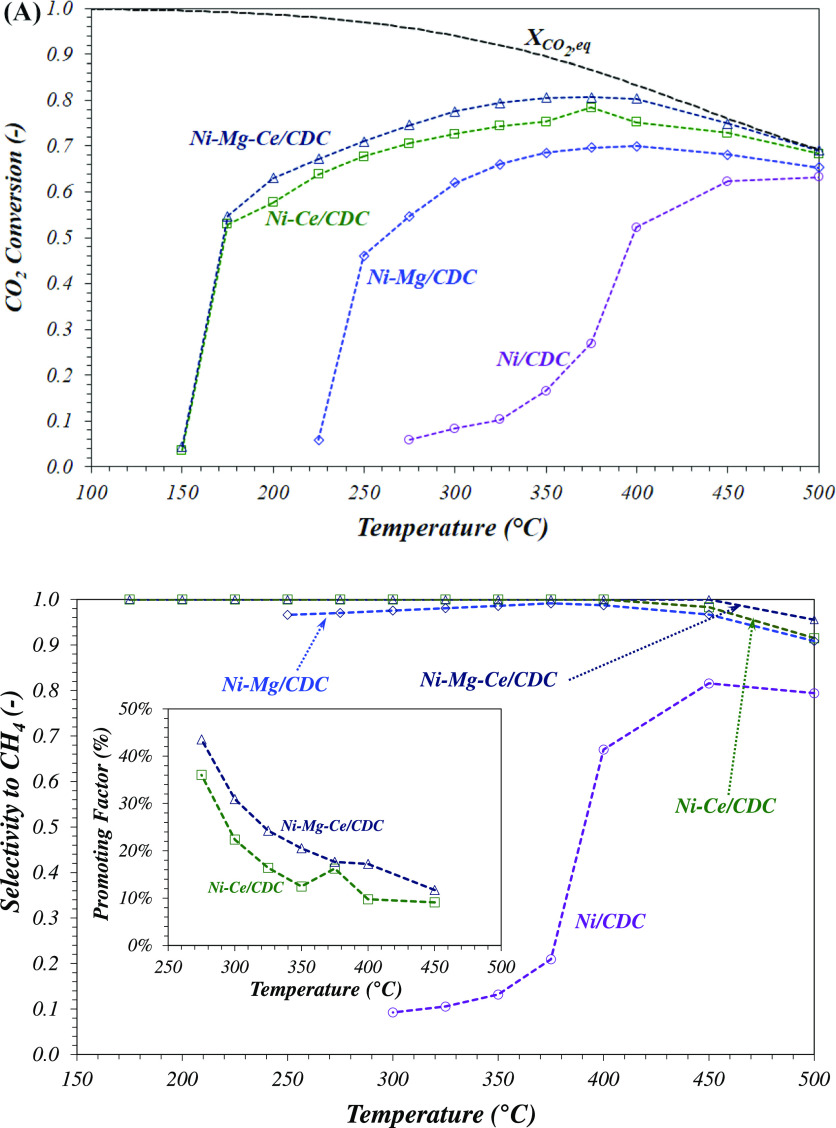
(A) Evolution of CO_2_ conversion with the reaction temperature, at 3.61 mol of CO_2_ g^–1^ of Ni h^–1^ and H_2_/CO_2_/N_2_ = 4:1:2. (B) Influence of the reaction temperature on the selectivity to CH_4_ and the promoting factor (inset).

The catalytic activity of the samples was evaluated at different temperatures maintaining the ratio of H_2_/CO_2_ = 4, and a spatial velocity referred to the Ni content of the catalysts of 3.61 mol of CO_2_ g^–1^ of Ni h^–1^. The influence of the reaction temperature in CO_2_ conversion and selectivity to CH_4_ is shown in panels A and B of Figure 7, respectively. The thermodynamic equilibrium data of CO_2_ conversion have also been included (dashed black line in Figure 7A).

As is well-known, the CO_2_ methanation reaction is thermodynamically limited at high temperatures, with the equilibrium values of CO_2_ conversion and CH_4_ selectivity, for example, being 69 and 89%, respectively, at 500 °C, which means a methane yield of 61.4%. In our case, these values are only attained by the Ce-promoted samples. As the reaction temperature decreases, thermodynamics favors the increase of the CO_2_ conversion; nevertheless, the kinetic limitations become more important. As a consequence, the conversions obtained at low temperatures are far from equilibrium. In any case, the results in Figure 7A indicate that the activity of promoted catalysts, expressed as CO_2_ conversion, is higher than that of monometallic Ni/CDC. If we compare the effect of the studied promotors, the addition of Ce leads to a superior performance compared to Mg; nevertheless, their combination enhances the catalytic activity even more, which appears as a very efficient approach for the design of optimized catalysts for the CO_2_ methanation reaction.

With regard to the selectivity of the reaction and similar to the evolution of CO_2_ conversion, CH_4_ formation is thermodynamically limited at high temperatures, with 89% being the maximum possible value at 500 °C in the studied conditions. This equilibrium value increases until ca. 100% at temperatures below 400 °C. The values shown in Figure 7B are close to the equilibrium values in all of the studied temperature range. Both Ce-containing samples exhibit the greatest values, attaining almost a 100% selectivity to methane, and the Mg-doped catalyst shows 97% CH_4_ selectivity. In fact, in the case of Ni–Ce–Mg/CDC and Ni–Ce/CDC catalysts, there is not detected CO at the exit of the reactor at reaction temperatures below 400 °C. Considering that the carbon atom mass balance has a low experimental error, the CH_4_ selectivity must be around 100%.

Along the complete interval of reaction temperatures, the activity of the catalysts follows the order: Ni–Mg–Ce/CDC ≅ Ni–Ce/CDC ≫ Ni–Mg/CDC ≫ Ni/CDC. Ni–Mg–Ce/CDC shows the higher activity attaining a maximum CO_2_ conversion of 80% between 350 and 400 °C with 100% CH_4_ selectivity. Similar trends are obtained over Ni–Ce/CDC and Ni–Mg/CDC, showing the maximum conversions at 350–400 °C (78 and 70% conversion for Ni–Ce/CDC and Ni–Mg/CDC, respectively). Therefore, the addition of Mg and/or especially Ce as catalytic promotors is clearly beneficial in this type of metallic catalyst supported on cellulose-derived carbon. This increase in activity is furthermore completed with an increase of the CH_4_ selectivity in the case of the Ce-containing catalysts (see Figure 7B), setting an optimum promotional effect of Ce species in this type of catalyst.^10,11^

A quantitative comparison of the catalytic performance obtained with multi-metallic catalysts with respect to the Ni/CDC sample can be made using the so-called “promoting factor”,^24^ defined as the increase of the methane yield for the promoted catalyst divided by the methane yield of the non-promoted catalyst (Ni/CDC). In our case, as a result of the very low CH_4_ yields obtained with the Ni/CDC sample at temperatures below 300 °C, large values of this factor are obtained. Consequently, we decided to take as a reference sample the Ni–Mg/CDC catalyst, obtaining the “promoting factor of Ce” (inset of Figure 7B). The values of this promoting factor for Ce decrease with the temperature as a consequence of the thermodynamic limitations, but on the interval of interest, i.e., at *T* < 250 °C, the increase largely exceeded 50% of the methane yield.

The more relevant result in this study is the high conversion obtained at temperatures below 200 °C. Ni–Mg–Ce/CDC and Ni–Ce/CDC catalysts are already active at 175 °C, attaining ca. 54.7% CO_2_ conversion and nearly 100% CH_4_ selectivity, confirming that Ce is an excellent promoter of Ni-based catalysts in this reaction.^10,11^ In contrast, Ni–Mg/CDC is completely inactive at temperatures below 250 °C. These results observed over Ce-promoted catalysts are comparable to the best results obtained using Ru-based catalysts.^24^ From an energetic and economical viewpoint, these results open a very promising route for the optimization of the Sabatier process based on this type of catalyst.

These promotional effects are a consequence of a combination of the properties of the catalysts, mainly those related to the reducibility of Ni species, the concentration and strength of surface basic sites, the metal particle size, and the interactions established among Ni, Ce, and Mg components.^45^ In this sense, the high activity of Ce-promoted catalysts can be related to the lower particle size of the metallic NPs obtained in the presence of CeO_2_ (see histograms in Figure 5), which is decisive for this structure-sensitive reaction.^9,56^ On the other hand, the oxygen vacancies in the CeO_2_ lattice may act as adsorption sites for CO_2_ molecules, thus boosting its further transformation.^14,15^ Moreover, the addition of appropriate amounts of Mg has several positive effects on the activity of the catalyst as a consequence of the increase of the number of basic sites (see Figure 4), increasing around 5% CO_2_ conversion using Ni–Mg–Ce/CDC in comparison to Ni–Ce/CDC in the entire temperature range. This improvement is due to both the diminution of the number of large Ni particles (see panels B and C of Figure 5), which contributes a lower turnover frequency (TOF),^57−59^ and the generation of proper basic sites (Figure 4), which likewise improves the CO_2_ adsorption, facilitating its subsequence conversion.^60^

Vogt et al.^57,58^ stated that the optimum Ni particle size should have enough terrace facets to supply hydrogen atoms and sufficient step sites with the highest hydrogenation activity. Moreover, the stage of direct dissociation of CO* to C* has the higher activation energy, which can be efficiently diminished by the addition of H to adsorbed CO*, which can be facilitated by the presence of reducible supports, such as CeO_2_. These, at the nickel–support interface, can increase the activation of CO* via, e.g., the addition of H* from the support.^58^

On the other side, although Mg enhances the activity and stability of the catalyst,^61,62^ high amounts of Mg could hinder the NiO reduction as a result of a higher interaction between NiO and MgO,^63^ as the TPR results suggest (Figure 3). Anyway, the presence of both O mobile from CeO_2_ and OH^–^ species adsorbed on the MgO surface seem to be beneficial to the formation and removal of water during the reaction. Indeed, some studies even consider these steps as the rate-determine steps in the CO_2_ reaction mechanism.^64,65^

**Figure 8:**
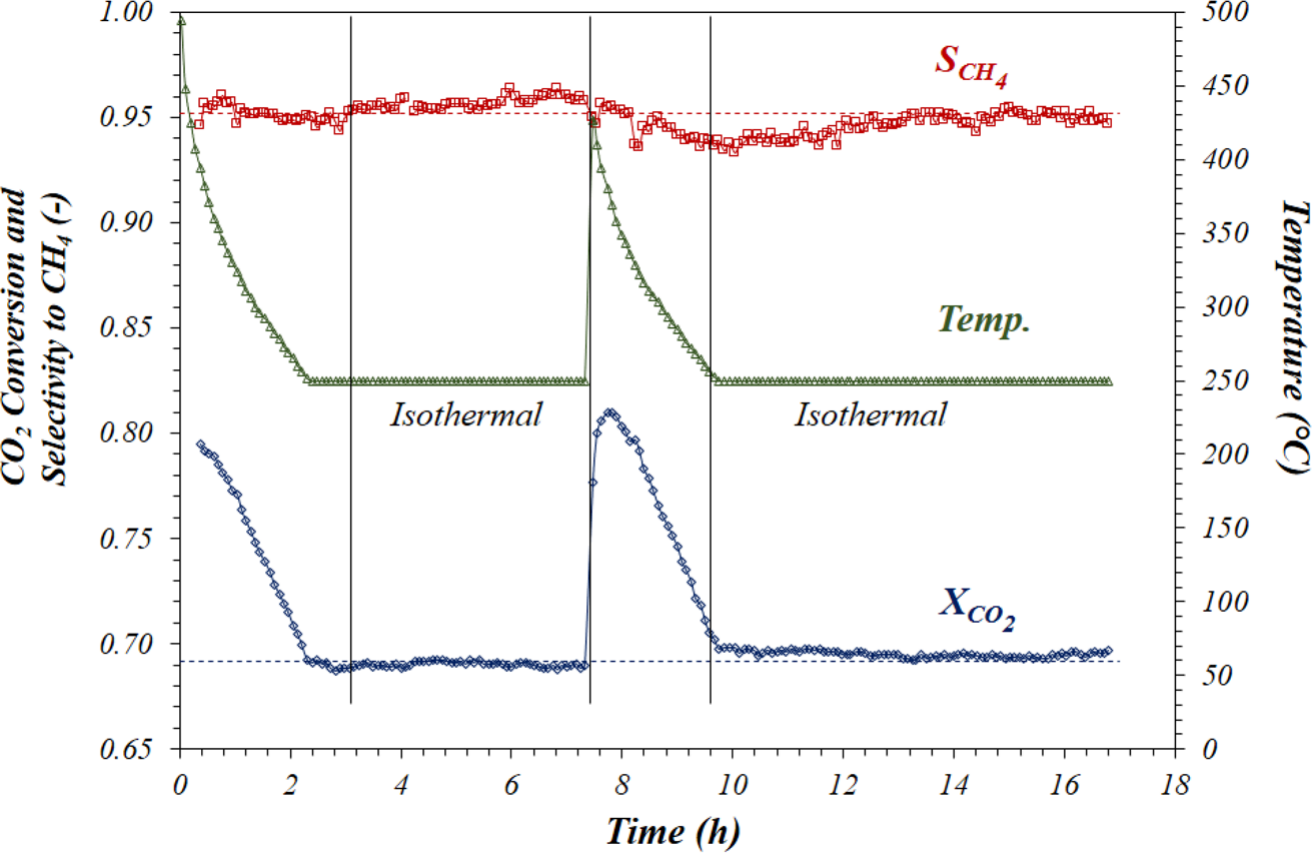
Stability test: evolution of CO_2_ conversion and selectivity to CH_4_ along time.

Finally, the potential stability of Ni–Mg–Ce/CDC was tested at 250 °C (Figure 8). In addition to CO_2_ conversion and CH_4_ selectivity, the temperature was likewise monitored and included. The results show that, when CO_2_ is introduced, after an operation cycle, the reaction starts and the temperature rises abruptly up to near 500 °C as a result of the elevated exothermicity of this reaction. After that, it decreases slowly until the target temperature, 250 °C. During the first isothermal period, both conversion and selectivity values (69 and 100%, respectively) are constant. This behavior is repeated at the second cycle, attaining the same constant values of conversion and selectivity after the stabilization period. CO was not detected at the exit of the reactor in any case.

All of these results indicate that, under the tested conditions, the catalyst is stable during the 12 h of isothermal experiments accumulated after the two cycles of reaction periods. However, despite these promising results, the real stability of this catalyst will be tested in much longer duration experiments.

**Figure 9:**
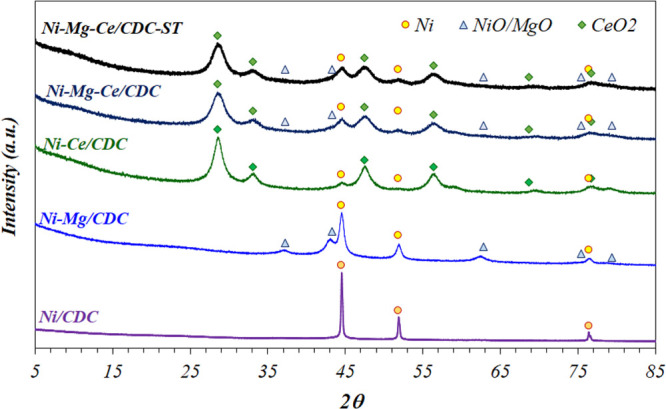
XRD patterns of catalysts after the activity test and Ni–Mg–Ce/CDC stability test (ST).

To check the catalyst state after the catalytic test, XRD analysis of spent catalysts was carried out (Figure 9). The average particle size values, calculated through the Scherrer equation, are 7, 6, and 5 nm for the Ni, NiO–MgO, and CeO_2_ phases, respectively, which are very similar to those results in the fresh catalyst (see Table 2). The results in Figure 9, in addition to HAADF and X-EDS (Figure 6), explain the stability observed during a total of 17 h (12 h under isothermal conditions at 250 °C). This is another decisive factor for the development of a promising CO_2_ methanation catalyst, which efficiently needs to operate in continuous mode.

## 4 Conclusion

One-pot Ce- and Mg-promoted Ni-based catalysts were successfully prepared by biomorphic mineralization, using cellulose as the carbon precursor of the support. The obtained samples exhibit strong metal–support interactions and great dispersion of the active phase on the carbonaceous support, even though presenting high metal loadings. Metal particles are in the nanoparticle range and seem to be responsible for the developed porosity of the catalyst support. In fact, the samples have pore diameters close to that of the particles in each case, which accounts for a clear metal effect on cellulose decomposition during the synthesis process, an effect that depends upon the promoter used in each case.

All of the catalysts show good performances in the CO_2_ methanation reaction working at atmospheric pressure and high spatial velocities (GHSV ranging from 3.0 × 10^4^ to 3.8 × 10^4^ h^–1^). Ce-containing catalysts reach the higher conversions, especially at low reaction temperatures, with just CH_4_ being detected as the reaction product at temperatures below 400 °C in all cases. The highest CO_2_ conversion is registered over the Ni–Mg–Ce/CDC sample in the entire temperature range, which somehow accounts for a kind of synergetic effect between Ce and Mg. Over this catalyst, a maximum of 80% conversion is reached between 350 and 400 °C. The most remarkable result is the high conversion obtained at temperatures below 200 °C over Ni–Mg–Ce/CDC and Ni–Ce/CDC catalysts, which are already active at 175 °C, attaining ca. 55% CO_2_ conversion and nearly 100% CH_4_ selectivity, confirming that Ce is an excellent promoter of Ni-based/CDC catalysts. Furthermore, the Ni–Mg–Ce/CDC sample shows great potential, also leading to a high methane yield. The optimum promotional effect of Ce is a consequence of a proper combination of the reducibility of Ni species, the specific concentration and strength of the surface basic sites, the stabilized metal particle size, and the strong interactions between Ni, Ce, and Mg components. In summary and considering energetic and economical aspects, the results obtained with these catalysts at a low temperature open a promising route for the optimization of the Sabatier process.

## Supporting Information Available

Complementary characterization results (TGA, Raman, and XPS) of fresh and used catalysts (PDF)

## References

[ref1] Thema M.; Bauer F.; Sterner M. Power-to-Gas: Electrolysis and Methanation Status Review. Renewable Sustainable Energy Rev. 2019, 112, 775–787. 10.1016/j.rser.2019.06.030.

[ref2] Bette N.; Thielemann J.; Schreiner M.; Mertens F. Methanation of CO_2_ over a (Mg,Al)O_*x*_ Supported Nickel Catalyst Derived from a (Ni,Mg,Al)-Hydrotalcite-like Precursor. ChemCatChem 2016, 8 (18), 2903–2906. 10.1002/cctc.201600469.

[ref3] Schaaf T.; Grünig J.; Schuster M. R.; Rothenfluh T.; Orth A. Methanation of CO_2_—Storage of Renewable Energy in a Gas Distribution System. Energy. Sustain. Soc. 2014, 4 (1), 1–14. 10.1186/s13705-014-0029-1.

[ref4] Kuznecova I.; Gusca J. Property Based Ranking of CO and CO_2_ Methanation Catalysts. Energy Procedia 2017, 128, 255–260. 10.1016/j.egypro.2017.09.068.

[ref5] Park J. N.; McFarland E. W. A Highly Dispersed Pd-Mg/SiO_2_ Catalyst Active for Methanation of CO_2_. J. Catal. 2009, 266 (1), 92–97. 10.1016/j.jcat.2009.05.018.

[ref6] Vannice M. A. The Catalytic Synthesis of Hydrocarbons from H_2_CO Mixtures over the Group VIII Metals. I. The Specific Activities and Product Distributions of Supported Metals. J. Catal. 1975, 37 (3), 449–461. 10.1016/0021-9517(75)90181-5.

[ref7] Rönsch S.; Schneider J.; Matthischke S.; Schlüter M.; Götz M.; Lefebvre J.; Prabhakaran P.; Bajohr S. Review on Methanation—From Fundamentals to Current Projects. Fuel 2016, 166, 276–296. 10.1016/j.fuel.2015.10.111.

[ref8] Ashok J.; Pati S.; Hongmanorom P.; Tianxi Z.; Junmei C.; Kawi S. A Review of Recent Catalyst Advances in CO_2_ Methanation Processes. Catal. Today 2020, 356, 471–489. 10.1016/j.cattod.2020.07.023.

[ref9] Hatzisymeon M.; Petala A.; Panagiotopoulou P. Carbon Dioxide Hydrogenation over Supported Ni and Ru Catalysts. Catal. Lett. 2021, 151 (3), 888–900. 10.1007/s10562-020-03355-0.

[ref10] Tada S.; Shimizu T.; Kameyama H.; Haneda T.; Kikuchi R. Ni/CeO_2_ Catalysts with High CO_2_ Methanation Activity and High CH_4_ Selectivity at Low Temperatures. Int. J. Hydrogen Energy 2012, 37 (7), 5527–5531. 10.1016/j.ijhydene.2011.12.122.

[ref11] Martin N. M.; Velin P.; Skoglundh M.; Bauer M.; Carlsson P. A. Catalytic Hydrogenation of CO_2_ to Methane over Supported Pd, Rh and Ni Catalysts. Catal. Sci. Technol. 2017, 7 (5), 1086–1094. 10.1039/C6CY02536F.

[ref12] Fukuhara C.; Hayakawa K.; Suzuki Y.; Kawasaki W.; Watanabe R. A Novel Nickel-Based Structured Catalyst for CO_2_ Methanation: A Honeycomb-Type Ni/CeO_2_ Catalyst to Transform Greenhouse Gas into Useful Resources. Appl. Catal., A 2017, 532, 12–18. 10.1016/j.apcata.2016.11.036.

[ref13] Hu F.; Tong S.; Lu K.; Chen C. M.; Su F. Y.; Zhou J.; Lu Z. H.; Wang X.; Feng G.; Zhang R. Reduced Graphene Oxide Supported Ni-Ce Catalysts for CO_2_ Methanation: The Support and Ceria Promotion Effects. J. CO2 Util. 2019, 34, 676–687. 10.1016/j.jcou.2019.08.020.

[ref14] Kim M. J.; Youn J. R.; Kim H. J.; Seo M. W.; Lee D.; Go K. S.; Lee K. B.; Jeon S. G. Effect of Surface Properties Controlled by Ce Addition on CO_2_ Methanation over Ni/Ce/Al_2_O_3_ Catalyst. Int. J. Hydrogen Energy 2020, 45 (46), 24595–24603. 10.1016/j.ijhydene.2020.06.144.

[ref15] Makdee A.; Chanapattharapol K. C.; Kidkhunthod P.; Poo-Arporn Y.; Ohno T. The Role of Ce Addition in Catalytic Activity Enhancement of TiO_2_-Supported Ni for CO_2_ methanation Reaction. RSC Adv. 2020, 10 (45), 26952–26971. 10.1039/D0RA04934D. 35515790 PMC9055534

[ref16] Zhang J.; Yang Y.; Liu J.; Xiong B. Mechanistic Understanding of CO_2_ Hydrogenation to Methane over Ni/ CeO_2_ Catalyst. Appl. Surf. Sci. 2021, 558, 149866 10.1016/j.apsusc.2021.149866.

[ref17] Aziz M. A. A.; Jalil A. A.; Triwahyono S.; Mukti R. R.; Taufiq-Yap Y. H.; Sazegar M. R. Highly Active Ni-Promoted Mesostructured Silica Nanoparticles for CO_2_ Methanation. Appl. Catal., B 2014, 147, 359–368. 10.1016/j.apcatb.2013.09.015.

[ref18] Lee H.; Kim J.; Lee D. A New Design and Synthesis Approach of Supported Metal Catalysts via Interfacial Hydrothermal-Oxidation/Reductive-Exolution Chemistry of Al Metal Substrate. Appl. Catal., A 2020, 594, 117461 10.1016/j.apcata.2020.117461.

[ref19] Bacariza M. C.; Graça I.; Bebiano S. S.; Lopes J. M.; Henriques C. Micro- and Mesoporous Supports for CO_2_ Methanation Catalysts: A Comparison between SBA-15, MCM-41 and USY Zeolite. Chem. Eng. Sci. 2018, 175, 72–83. 10.1016/j.ces.2017.09.027.

[ref20] Swalus C.; Jacquemin M.; Poleunis C.; Bertrand P.; Ruiz P. CO_2_ Methanation on Rh/γ-Al_2_O_3_ Catalyst at Low Temperature: “In Situ” Supply of Hydrogen by Ni/Activated Carbon Catalyst. Appl. Catal., B 2012, 125, 41–50. 10.1016/j.apcatb.2012.05.019.

[ref21] Wang X.; Liu Y.; Zhu L.; Li Y.; Wang K.; Qiu K.; Tippayawong N.; Aggarangsi P.; Reubroycharoen P.; Wang S. Biomass Derived N-Doped Biochar as Efficient Catalyst Supports for CO_2_ Methanation. J. CO2 Util. 2019, 34, 733–741. 10.1016/j.jcou.2019.09.003.

[ref22] O’byrne J. P.; Owen R. E.; Minett D. R.; Pascu S. I.; Plucinski P. K.; Jones M. D.; Mattia D. High CO_2_ and CO Conversion to Hydrocarbons Usingbridged Fe Nanoparticles on Carbon Nanotubes. Catal. Sci. Technol. 2013, 3 (1), 1202 10.1039/c3cy20854k.

[ref23] De S.; Balu A. M.; Van Der Waal J. C.; Luque R. Biomass-Derived Porous Carbon Materials: Synthesis and Catalytic Applications. ChemCatChem 2015, 7 (11), 1608–1629. 10.1002/cctc.201500081.

[ref24] Lee W. J.; Li C.; Prajitno H.; Yoo J.; Patel J.; Yang Y.; Lim S. Recent Trend in Thermal Catalytic Low Temperature CO_2_ Methanation: A Critical Review. Catal. Today 2021, 368, 2–19. 10.1016/j.cattod.2020.02.017.

[ref25] Sankaranarayanan S.; Lakshmi D. S.; Vivekanandhan S.; Ngamcharussrivichai C. Biocarbons as Emerging and Sustainable Hydrophobic/Oleophilic Sorbent Materials for Oil/Water Separation. Sustain. Mater. Technol. 2021, 28, e00268 10.1016/j.susmat.2021.e00268.

[ref26] Santhiago M.; Garcia P. S.; Strauss M. Bio-Based Nanostructured Carbons toward Sustainable Technologies. Curr. Opin. Green Sustain. Chem. 2018, 12, 22–26. 10.1016/j.cogsc.2018.04.009.

[ref27] Fan T.-X.; Chow S.-K.; Zhang D. Biomorphic Mineralization: From Biology to Materials. Prog. Mater. Sci. 2009, 54, 542–659. 10.1016/j.pmatsci.2009.02.001.

[ref28] Lam E.; Luong J. H. T. Carbon Materials as Catalyst Supports and Catalysts in the Transformation of Biomass to Fuels and Chemicals. ACS Catal. 2014, 4 (10), 3393–3410. 10.1021/cs5008393.

[ref29] Hoekstra J.; Versluijs-Helder M.; Vlietstra E. J.; Geus J. W.; Jenneskens L. W. Carbon-Supported Base Metal Nanoparticles: Cellulose at Work. ChemSusChem 2015, 8 (6), 985–989. 10.1002/cssc.201403364. 25704034

[ref30] Singh M.; Yee B. M. Reactive Processing of Environmentally Conscious, Biomorphic Ceramics from Natural Wood Precursors. J. Eur. Ceram. Soc. 2004, 24 (2), 209–217. 10.1016/S0955-2219(03)00244-9.

[ref31] Cazaña F.; Latorre N.; Tarifa P.; Labarta J.; Romeo E.; Monzón A. Synthesis of Graphenic Nanomaterials by Decomposition of Methane on a Ni-Cu/Biomorphic Carbon Catalyst. Kinetic and Characterization Results. Catal. Today 2018, 299, 67–79. 10.1016/j.cattod.2017.03.056.

[ref32] Henao W.; Cazaña F.; Tarifa P.; Romeo E.; Latorre N.; Sebastian V.; Delgado J. J.; Monzón A. Selective Synthesis of Carbon Nanotubes by Catalytic Decomposition of Methane Using Co-Cu/Cellulose Derived Carbon Catalysts: A Comprehensive Kinetic Study. Chem. Eng. J. 2021, 404, 126103 10.1016/j.cej.2020.126103.

[ref33] Azuara M.; Latorre N.; Villacampa J. I.; Sebastian V.; Cazaña F.; Romeo E.; Monzón A. Use of Ni Catalysts Supported on Biomorphic Carbon Derived from Lignocellulosic Biomass Residues in the Decomposition of Methane. Front. Energy Res. 2019, 7 (MAR), 34 10.3389/fenrg.2019.00034.

[ref34] Cazaña F.; Jimaré M. T.; Romeo E.; Sebastián V.; Irusta S.; Latorre N.; Royo C.; Monzón A. Kinetics of Liquid Phase Cyclohexene Hydrogenation on Pd-Al/Biomorphic Carbon Catalysts. Catal. Today 2015, 249, 127–136. 10.1016/j.cattod.2014.11.022.

[ref35] Cazaña F.; Galetti A.; Meyer C.; Sebastián V.; Centeno M. A.; Romeo E.; Monzón A. Synthesis of Pd-Al/Biomorphic Carbon Catalysts Using Cellulose as Carbon Precursor. Catal. Today 2018, 301, 226–238. 10.1016/j.cattod.2017.05.026.

[ref36] Santos J. L.; Mäki-Arvela P.; Wärnå J.; Monzón A.; Centeno M. A.; Murzin D. Y. Hydrodeoxygenation of Vanillin over Noble Metal Catalyst Supported on Biochars: Part II: Catalytic Behaviour. Appl. Catal., B 2020, 268, 118425 10.1016/j.apcatb.2019.118425.

[ref37] Santos J. L.; Mäki-Arvela P.; Monzón A.; Murzin D. Y.; Centeno M. Á. Metal Catalysts Supported on Biochars: Part I Synthesis and Characterization. Appl. Catal., B 2020, 268, 118423 10.1016/j.apcatb.2019.118423.

[ref38] Zwinkels M. F. M.; JÄRÅS S. G.; Menon P. G.; Griffin T. A. Catalytic Materials for High-Temperature Combustion. Catal. Rev.: Sci. Eng. 1993, 35 (3), 319–358. 10.1080/01614949308013910.

[ref39] Centeno M.; Ramírez Reina T.; Ivanova S.; Laguna O.; Odriozola J. Au/CeO_2_ Catalysts: Structure and CO Oxidation Activity. Catalysts 2016, 6 (10), 158 10.3390/catal6100158.

[ref40] Moliner R.; Echegoyen Y.; Suelves I.; Lázaro M. J.; Palacios J. M. Ni-Mg and Ni-Cu-Mg Catalysts for Simultaneous Production of Hydrogen and Carbon Nanofibers. The Effect of Calcination Temperature. Int. J. Hydrogen Energy 2008, 33 (6), 1719–1728. 10.1016/j.ijhydene.2008.01.005.

[ref41] Scherdel C.; Reichenauer G.; Wiener M. Relationship between Pore Volumes and Surface Areas Derived from the Evaluation of N2-Sorption Data by DR-, BET- and t-Plot. Microporous Mesoporous Mater. 2010, 132 (3), 572–575. 10.1016/j.micromeso.2010.03.034.

[ref42] Bitter J. H.; Van Der Lee M. K.; Slotboom A. G. T.; Van Dillen A. J.; De Jong K. P. Synthesis of Highly Loaded Highly Dispersed Nickel on Carbon Nanofibers by Homogeneous Deposition-Precipitation. Catal. Lett. 2003, 89 (1–2), 139–142. 10.1023/A:1024744131630.

[ref43] Nieto-Márquez A.; Gil S.; Romero A.; Valverde J. L.; Gómez-Quero S.; Keane M. A. Gas Phase Hydrogenation of Nitrobenzene over Acid Treated Structured and Amorphous Carbon Supported Ni Catalysts. Appl. Catal., A 2009, 363 (1–2), 188–198. 10.1016/j.apcata.2009.05.016.

[ref44] Ha Q. L. M.; Armbruster U.; Atia H.; Schneider M.; Lund H.; Agostini G.; Radnik J.; Vuong H. T.; Martin A. Development of Active and Stable Low Nickel Content Catalysts for Dry Reforming of Methane. Catalysts 2017, 7 (5), 157 10.3390/catal7050157.

[ref45] Siakavelas G. I.; Charisiou N. D.; AlKhoori S.; AlKhoori A. A.; Sebastian V.; Hinder S. J.; Baker M. A.; Yentekakis I. V.; Polychronopoulou K.; Goula M. A. Highly Selective and Stable Nickel Catalysts Supported on Ceria Promoted with Sm_2_O_3_, Pr_2_O_3_ and MgO for the CO_2_ Methanation Reaction. Appl. Catal., B 2021, 282, 119562 10.1016/j.apcatb.2020.119562.

[ref46] Hwan Lee Y.; Yoon Ahn J.; Duc Nguyen D.; Woong Chang S.; Su Kim S.; Moon Lee S. Role of Oxide Support in Ni Based Catalysts for CO_2_ Methanation. RSC Adv. 2021, 11 (29), 17648–17657. 10.1039/D1RA02327F. 35480170 PMC9036400

[ref47] Ren J.; Mebrahtu C.; Palkovits R. Ni-Based Catalysts Supported on Mg-Al Hydrotalcites with Different Morphologies for CO_2_ Methanation: Exploring the Effect of Metal-Support Interaction. Catal. Sci. Technol. 2020, 10 (6), 1902–1913. 10.1039/C9CY02523E.

[ref48] Zhao K.; Wang W.; Li Z. Highly Efficient Ni/ZrO_2_ Catalysts Prepared via Combustion Method for CO_2_ Methanation. J. CO2 Util. 2016, 16, 236–244. 10.1016/j.jcou.2016.07.010.

[ref49] Pan Q.; Peng J.; Sun T.; Wang S.; Wang S. Insight into the Reaction Route of CO_2_ Methanation: Promotion Effect of Medium Basic Sites. Catal. Commun. 2014, 45, 74–78. 10.1016/j.catcom.2013.10.034.

[ref50] Bette N.; Thielemann J.; Schreiner M.; Mertens F. Methanation of CO_2_ over a(Mg,Al)O_*x*_ SupportedN Ickel Catalyst Derived from a (Ni,Mg,Al)-Hydrotalcite-like Precursor. ChemCatChem 2016, 8 (18), 2903–2906. 10.1002/cctc.201600469.

[ref51] He L.; Lin Q.; Liu Y.; Huang Y. Unique Catalysis of Ni-Al Hydrotalcite Derived Catalyst in CO_2_ Methanation: Cooperative Effect between Ni Nanoparticles and a Basic Support. J. Energy Chem. 2014, 23 (5), 587–592. 10.1016/S2095-4956(14)60144-3.

[ref52] Aldana P. A. U.; Ocampo F.; Kobl K.; Louis B.; Thibault-Starzyk F.; Daturi M.; Bazin P.; Thomas S.; Roger A. C. Catalytic CO_2_ Valorization into CH4 on Ni-Based Ceria-Zirconia. Reaction Mechanism by Operando IR Spectroscopy. Catal. Today 2013, 215, 201–207. 10.1016/j.cattod.2013.02.019.

[ref53] AlKetbi M.; Polychronopoulou K.; Abi Jaoude M.; Vasiliades M. A.; Sebastian V.; Hinder S. J.; Baker M. A.; Zedan A. F.; Efstathiou A. M. Cu-Ce-La-O_x_ as Efficient CO Oxidation Catalysts: Effect of Cu Content. Appl. Surf. Sci. 2020, 505, 144474 10.1016/j.apsusc.2019.144474.

[ref54] Mahendiran C.; Maiyalagan T.; Scott K.; Gedanken A. Synthesis of a Carbon-Coated NiO/MgO Core/Shell Nanocomposite as a Pd Electro-Catalyst Support for Ethanol Oxidation. Mater. Chem. Phys. 2011, 128 (3), 341–347. 10.1016/j.matchemphys.2011.02.067.

[ref55] Cazzanelli E.; Kuzmin A.; Mariotto G.; Mironova-Ulmane N. Study of Vibrational and Magnetic Excitations in Ni_*c*_Mg_1–*c*_O Solid Solutions by Raman Spectroscopy. J. Phys.: Condens. Matter 2003, 15 (12), 2045–2052. 10.1088/0953-8984/15/12/321.

[ref56] Lin L.; Gerlak C. A.; Liu C.; Llorca J.; Yao S.; Rui N.; Zhang F.; Liu Z.; Zhang S.; Deng K.; Murray C. B.; Rodriguez J. A.; Senanayake S. D. Effect of Ni Particle Size on the Production of Renewable Methane from CO_2_ over Ni/CeO_2_ Catalyst. J. Energy Chem. 2021, 61, 602 10.1016/j.jechem.2021.02.021.

[ref57] Vogt C.; Groeneveld E.; Kamsma G.; Nachtegaal M.; Lu L.; Kiely C. J.; Berben P. H.; Meirer F.; Weckhuysen B. M. Unravelling Structure Sensitivity in CO_2_ Hydrogenation over Nickel. Nat. Catal. 2018, 1 (2), 127–134. 10.1038/s41929-017-0016-y.

[ref58] Vogt C.; Monai M.; Sterk E. B.; Palle J.; Melcherts A. E. M.; Zijlstra B.; Groeneveld E.; Berben P. H.; Boereboom J. M.; Hensen E. J. M.; Meirer F.; Filot I. A. W.; Weckhuysen B. M. Understanding Carbon Dioxide Activation and Carbon–Carbon Coupling over Nickel. Nat. Commun. 2019, 10 (1), 1–10. 10.1038/s41467-019-12858-3. 31767838 PMC6877608

[ref59] Hao Z.; Shen J.; Lin S.; Han X.; Chang X.; Liu J.; Li M.; Ma X. Decoupling the Effect of Ni Particle Size and Surface Oxygen Deficiencies in CO_2_ Methanation over Ceria Supported Ni. Appl. Catal., B 2021, 286, 119922 10.1016/j.apcatb.2021.119922.

[ref60] Guo M.; Lu G. The Effect of Impregnation Strategy on Structural Characters and CO_2_ Methanation Properties over MgO Modified Ni/SiO_2_ Catalysts. Catal. Commun. 2014, 54, 55–60. 10.1016/j.catcom.2014.05.022.

[ref61] Liu J.; Bing W.; Xue X.; Wang F.; Wang B.; He S.; Zhang Y.; Wei M. Alkaline-Assisted Ni Nanocatalysts with Largely Enhanced Low-Temperature Activity toward CO_2_ Methanation. Catal. Sci. Technol. 2016, 6, 3976 10.1039/C5CY02026C.

[ref62] Fan W. K.; Tahir M. Recent Trends in Developments of Active Metals and Heterogenous Materials for Catalytic CO_2_ Hydrogenation to Renewable Methane: A Review. J. Environ. Chem. Eng. 2021, 9, 105460 10.1016/j.jece.2021.105460.

[ref63] Völs P.; Hilbert S.; Störr B.; Bette N.; Lißner A.; Seidel J.; Mertens F. Methanation of CO_2_ and CO by (Ni,Mg,Al)-Hydrotalcite-Derived and Related Catalysts with Varied Magnesium and Aluminum Oxide Contents. Ind. Eng. Chem. Res. 2021, 60, 5114–5123. 10.1021/acs.iecr.1c00028.

[ref64] Cárdenas-Arenas A.; Quindimil A.; Davó-Quiñonero A.; Bailón-García E.; Lozano-Castelló D.; De-La-Torre U.; Pereda-Ayo B.; González-Marcos J. A.; González-Velasco J. R.; Bueno-López A. Isotopic and in Situ DRIFTS Study of the CO_2_ Methanation Mechanism Using Ni/CeO_2_ and Ni/Al_2_O_3_ Catalysts. Appl. Catal., B 2020, 265, 118538 10.1016/j.apcatb.2019.118538.

[ref65] Huang J.; Li X.; Wang X.; Fang X.; Wang H.; Xu X. New Insights into CO_2_ Methanation Mechanisms on Ni/MgO Catalysts by DFT Calculations: Elucidating Ni and MgO Roles and Support Effects. J. CO2 Util. 2019, 33 (April), 55–63. 10.1016/j.jcou.2019.04.022.

