# Supplementary material for: Highly Active Ce- and Mg-Promoted Ni Catalysts Supported on Cellulose-Derived Carbon for Low-Temperature CO2 Methanation
Source: Energy Fuels. 2021 Sep 1;35(21):17212–24. doi: 10.1021/acs.energyfuels.1c01682 (PMC9161724; doi:10.1021/acs.energyfuels.1c01682)
Supplement: Supplementary file 1 — ef1c01682_si_001.pdf [file ef1c01682_si_001.pdf]

Highly Active Ce and Mg Promoted Ni Catalysts Supported on Cellulose Derived Carbon for Low Temperature CO_2 Methanation.

Pilar Tarifa, Cristina Megías-Sayago, Fernando Cazaña, Miguel González-Martín, Nieves Latorre, Eva Romeo, Juan José Delgado[†]*, Antonio Monzón*[*]*.*

Department of Chemical and Environmental Engineering. Institute of Nanoscience and Materials of Aragón (INMA). University of Zaragoza. Spain.

[†]Department of Materials Science, Metallurgical Engineering and Inorganic Chemistry, University of Cádiz, E-11510 Puerto Real, Spain

Number of pages: 5

Number of Tables: 1

Number of Figures: 3

List of Figures.

Figure S.I.-1. TGA-air results of the fresh catalysts.

Figure S.I.-2. Raman Spectra of: (A) fresh catalysts; (B) used catalysts; (C) used catalyst, zone (300-700 cm^{-1}) of the used catalysts.

Figure S.I.-3. XPS core level spectra of fresh catalysts: (A) C1s; (B) Ni $2p_{3/2}$; (C) Ce 3d; (D) Mg 1s; (E) O 1s.

S.I. Figures

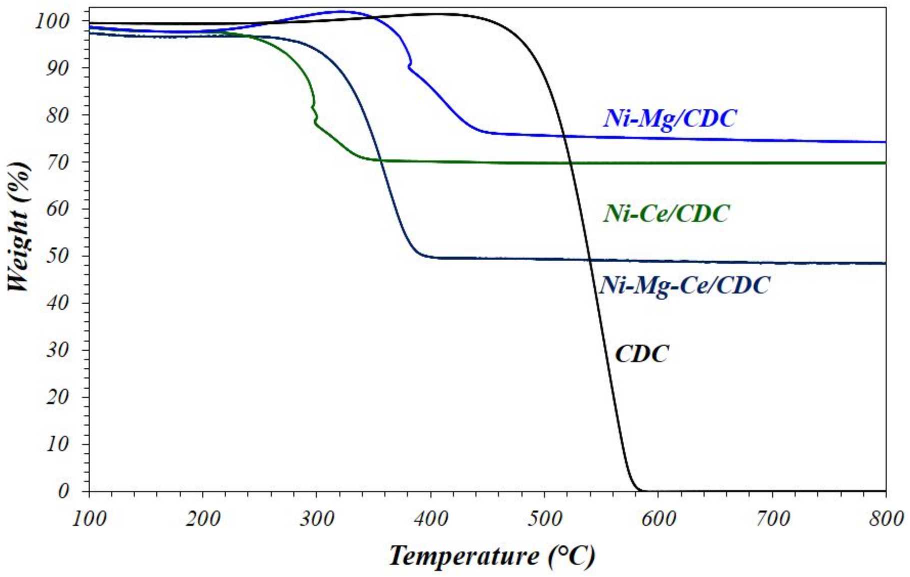

Figure S.I.-1. TGA-air results of the fresh catalysts.

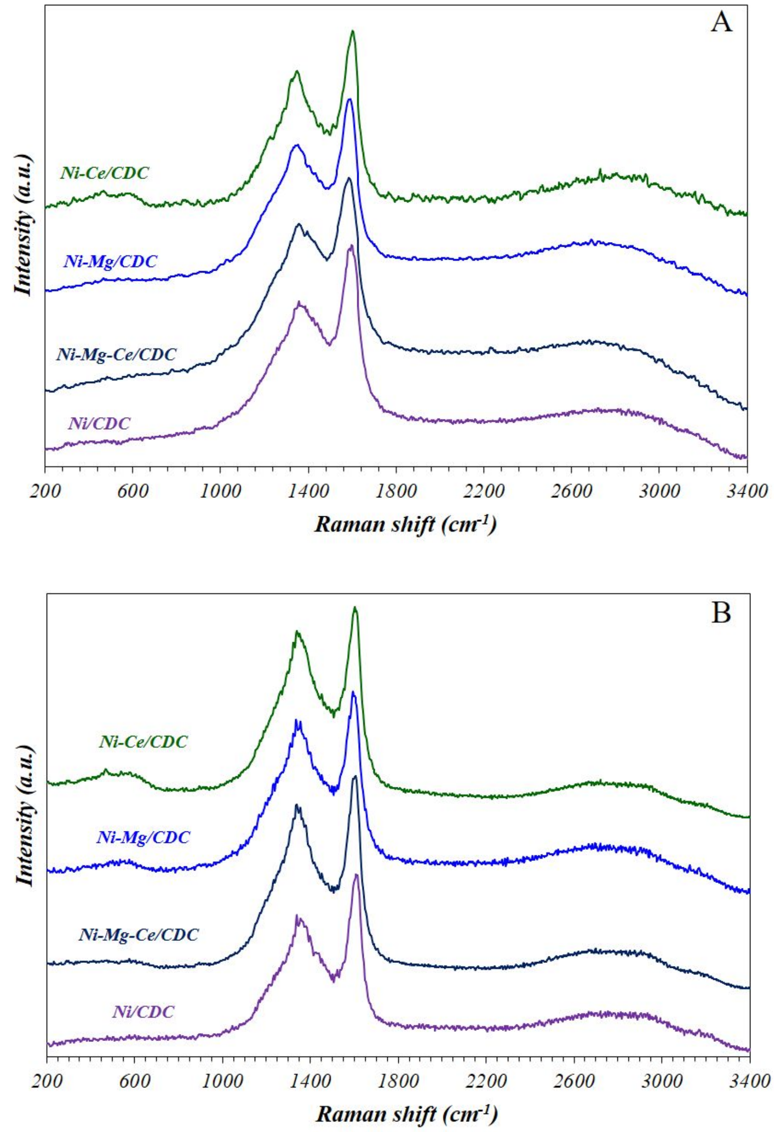

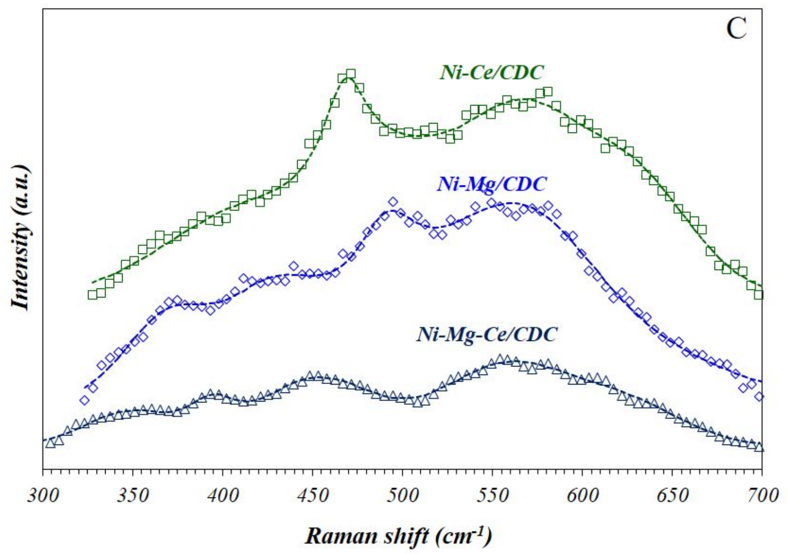

Figure S.I.-2. Raman Spectra of: (A) fresh catalysts; (B) used catalysts; (C) used catalyst, zone (300-700 cm^{-1}) of the used catalysts.

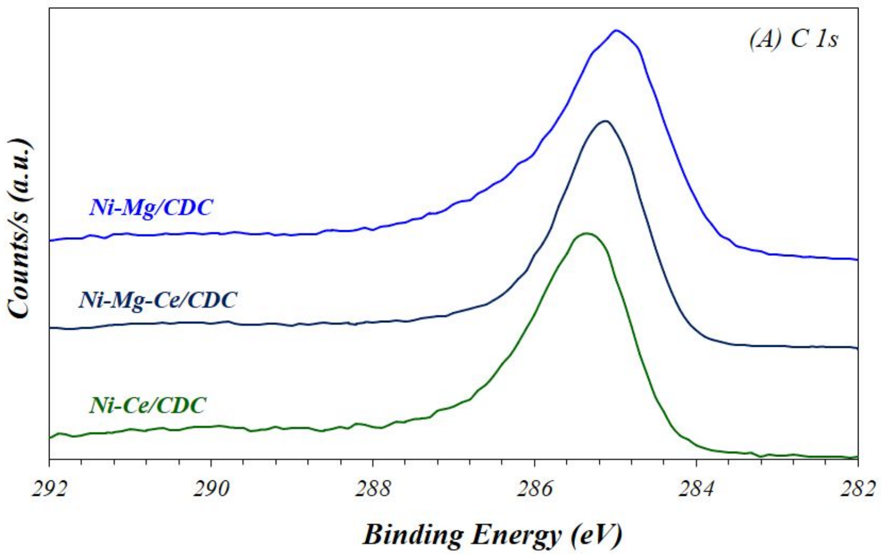

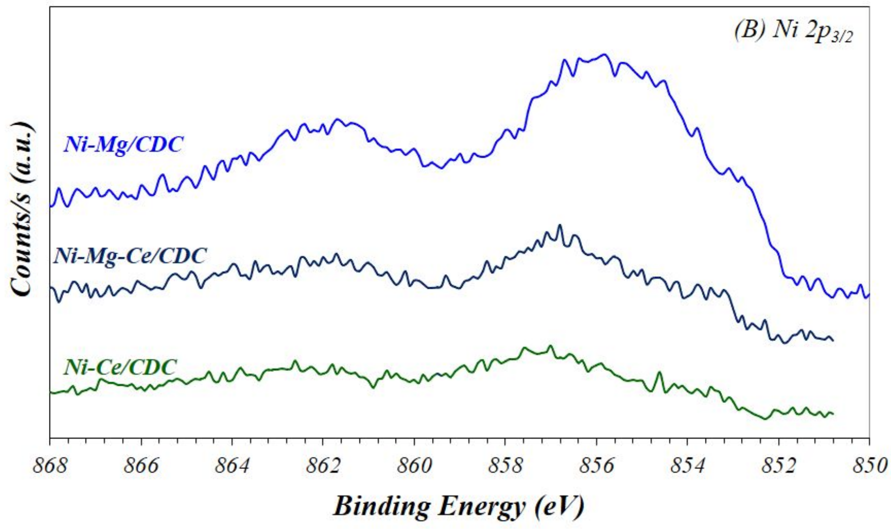

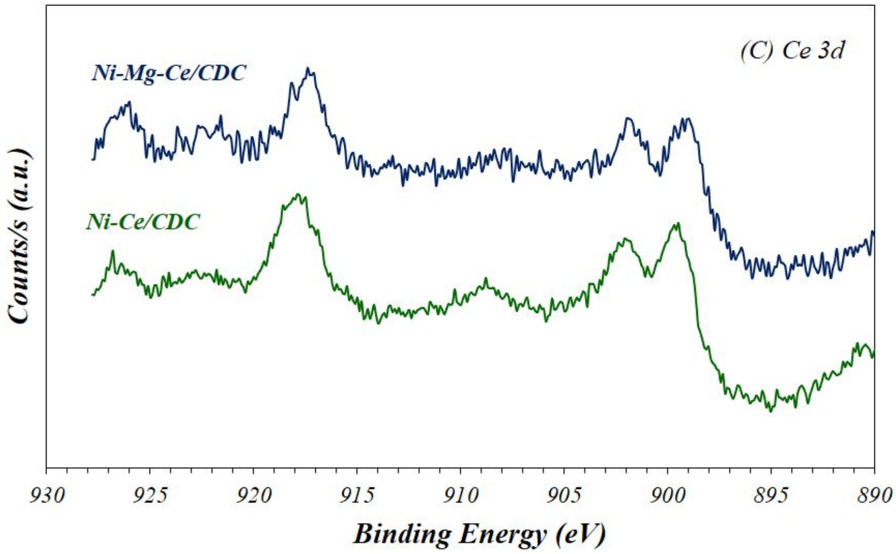
(C) Ce 3d
Ni-Mg-Ce/CDC
Ni-Ce/CDC
Counts/s (a.u.)
930
925
920
915
910
905
900
895
890
Binding Energy (eV)

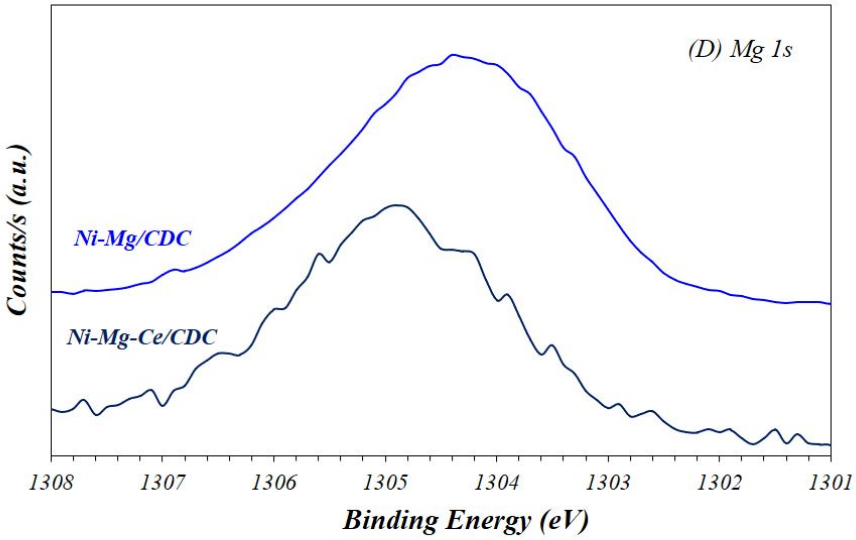
(D) Mg 1s
Ni-Mg/CDC
Ni-Mg-Ce/CDC
Counts/s (a.u.)
1308
1307
1306
1305
1304
1303
1302
1301
Binding Energy (eV)

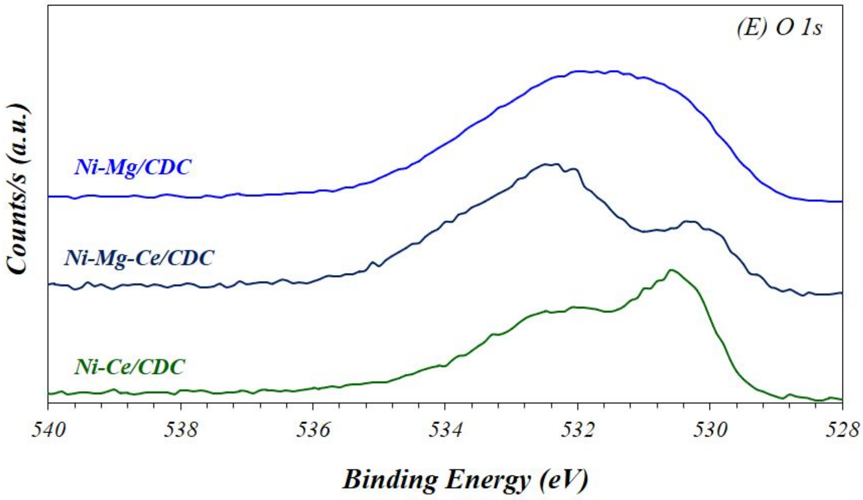
(E) O 1s
Ni-Mg/CDC
Ni-Mg-Ce/CDC
Ni-Ce/CDC
Counts/s (a.u.)
540
538
536
534
532
530
528
Binding Energy (eV)

Figure S.I.-3. XPS core level spectra of fresh catalysts: (A) C1s; (B) Ni $2p_{3/2}$; (C) Ce 3d; (D) Mg 1s; (E) O 1s.

Table S.I.-1. XPS results of the fresh samples.

Catalyst		*Ni 2p*		*Mg 1s*	*Ce 3d*	*C 1s*				*O 1s*		*Ni/Mg/Ce*
	specie	***Ni (0)***	***Ni (II)***			***C-C***	***C-O***	***C=O***	***C-OOH***	***O-Me***	***O-C***	
NiMg/CDC	***B. E. (eV)***	*852.8*	*855.8*	*1304.4*	*-*	*284.9*	*286.1*	*287.1*	*288.5*	*530.6*	*532.3*	*1/3.96/0*
	%	*3.1*		*12.3*	*0*	*59.6*				*25.0*		
	%	*3*	*97*		*0*	*71*	*16*	*7*	*3*	*22*	*78*	
NiMgCe/CDC	***B. E. (eV)***	*853.5*	*856.5*	*1304.8*	*883.1*	*285.1*	*286.0*	*287.1*	*288.5*	*530.1*	*532.5*	*1/3.72/0.45*
	%	*1.1*		*4.1*	*0.5*	*77.3*				*17.0*		
	%	*10.6*	*89.4*			*71.9*	*13.5*	*4.4*	*5.3*	*12*	*88*	
NiCe/CDC	***B. E. (eV)***	*853.8*	*857.0*	*-*	*883.8*	*285.2*	*286.0*	*287.1*	*288.5*	*530.5*	*532.1*	*1/0/1.03*
	%	*2.6*		*0*	*2.7*	*71.2*				*23.5*		
	%	*9.1*	*90.9*	*0*		*58.3*	*28.1*	*6.2*	*3.6*	*28.7*	*71.3*	
